# False signals induced by single-cell imputation

**DOI:** 10.12688/f1000research.16613.2

**Published:** 2019-03-05

**Authors:** Tallulah S. Andrews, Martin Hemberg

**Affiliations:** 1 Wellcome Trust Sanger Institute, Hinxton, Cambridgeshire, CB10 1SA, UK

**Keywords:** Gene expression, single-cell, RNA-seq, Imputation, Type 1 errors, Reproducibility

## Abstract

**Background:** Single-cell RNA-seq is a powerful tool for measuring gene expression at the resolution of individual cells.  A challenge in the analysis of this data is the large amount of zero values, representing either missing data or no expression. Several imputation approaches have been proposed to address this issue, but they generally rely on structure inherent to the dataset under consideration they may not provide any additional information, hence, are limited by the information contained therein and the validity of their assumptions.

**Methods:** We evaluated the risk of generating false positive or irreproducible differential expression when imputing data with six different methods. We applied each method to a variety of simulated datasets as well as to permuted real single-cell RNA-seq datasets and consider the number of false positive gene-gene correlations and differentially expressed genes. Using matched 10X and Smart-seq2 data we examined whether cell-type specific markers were reproducible across datasets derived from the same tissue before and after imputation.

**Results:** The extent of false-positives introduced by imputation varied considerably by method. Data smoothing based methods, MAGIC, knn-smooth and dca, generated many false-positives in both real and simulated data. Model-based imputation methods typically generated fewer false-positives but this varied greatly depending on the diversity of cell-types in the sample. All imputation methods decreased the reproducibility of cell-type specific markers, although this could be mitigated by selecting markers with large effect size and significance.

**Conclusions: **Imputation of single-cell RNA-seq data introduces circularity that can generate false-positive results. Thus, statistical tests applied to imputed data should be treated with care. Additional filtering by effect size can reduce but not fully eliminate these effects. Of the methods we considered, SAVER was the least likely to generate false or irreproducible results, thus should be favoured over alternatives if imputation is necessary.

## Introduction

Single-cell RNA-seq (scRNA-seq) is a powerful technique for assaying the whole transcriptome at the resolution of individual cells. Although experimental protocols have evolved rapidly, there is still no strong consensus on how to best analyse the data. An important challenge to analysing scRNA-seq data is the high frequency of zero values, often referred to as dropouts, and the overall high levels of noise due to the low amounts of input RNA obtained from individual cells. Recently there have been four methods published ( Gong *et al*., 2018; Huang *et al*., 2018; Li & Li, 2018; van Dijk *et al*., 2018) which attempt to address these challenges though imputation, with several more under development ( Deng *et al*., 2018; Mongia *et al*., 2018; Moussa & Mandoiu, 2018; Wagner *et al*., 2017). Several recently introduced methods employ deep learning autoencoders for processing scRNA-seq data, including imputation and data-smoothing ( Eraslan *et al*., 2019; Hu & Greene, 2018; Wang & Gu, 2018; Wang *et al*., 2018).

Imputation is a common approach when dealing with sparse genomics data. A notable example has been the improvements to GWAS sensitivity and resolution when using haplotype information to impute unobserved SNPs ( Visscher *et al*., 2017). Unlike scRNA-seq data, this imputation employs an external reference dataset, often the 1000 Genomes project, to infer the missing values ( Chou *et al*., 2016). Such a reference does not yet exist for scRNA-seq data, and thus imputation methods can only use information internal to the dataset to be imputed. As a result there is a degree of circularity introduced into the dataset following imputation which could result in false positive results when identifying marker genes, gene-gene correlations, or testing differential expression. Zero values in scRNA-seq may arise due to low experimental sensitivity, e.g. sequencing sampling noise, technical dropouts during library preparation, or because biologically the gene is not expressed in the particular cell. Thus, one challenge when imputing expression values is to distinguish true zeros from missing values.

Many imputation methods, such as SAVER ( Huang *et al*., 2018), DrImpute ( Gong *et al*., 2018) and scImpute ( Li & Li, 2018), use models of the expected gene expression distribution to distinguish true biological zeros from zeros originating from technical noise. Because these gene expression distributions assume homogenous cell populations, they first identify clusters of similar cells to which an appropriate mixture model is fitted. Values falling above a given probability threshold to originate from technical effects are subsequently imputed. For example, scImpute models log-normalized expression values as a mixture of gamma-distributed dropouts and normally-distributed true observations. Alternatively some scRNA-seq imputation methods perform data smoothing. In contrast to imputation, which only attempt to infer values of missing data, smoothing reduces noise present in observed values by using information from neighbouring data points. Both MAGIC ( van Dijk *et al*., 2018) and knn-smooth ( Wagner *et al*., 2017) perform data smoothing for single-cell data using each cell’s k nearest neighbours either through the application of diffusion models or weighted sums respectively.

Previous benchmarking of these imputation methods was based on positive controls, i.e. the ability to recover true signals within noisy data ( Zhang & Zhang, 2018); the potential for false signals to be introduced into a dataset by these imputation methods was not considered, and it was concluded that most imputation methods provide a small improvement. We consider negative controls to evaluate the risks of introducing false positive when using imputation for single-cell datasets. Testing of the four published imputation methods, MAGIC, SAVER, scImpute, and DrImpute and one currently unpublished method, knn-smooth, revealed that all methods can introduce false positive signals into data. While some methods, performed well on simulated data, permuting real scRNA-seq data revealed high variability in performance on different datasets. We show that statistical tests applied to imputed data should be treated with care, and that results found in imputed data may not be reproducible across datasets.

## Methods

Six different single-cell RNASeq imputation methods were tested: SAVER ( Huang *et al*., 2018), DrImpute ( Gong *et al*., 2018), scImpute ( Li & Li, 2018), dca ( Eraslan *et al*., 2019), MAGIC ( van Dijk *et al*., 2018) and knn-smooth ( Wagner *et al*., 2017). These include all of the published imputation methods, at present, an additional data smoothing approach, knn-smooth, to contrast to the only published data smoothing method, MAGIC. We have also included a single autoencoder-based method, dca ( Eraslan *et al*., 2019). Unless specified otherwise these were run with default parameters ( Table 1). Each method was applied to either the raw-counts or log2 counts per million normalized data, as calculated scater ( McCarthy *et al*., 2017), as appropriate.

**Table 1.** Imputation methods.

Method	Model	Parameter(s)	Range	Reference
scImpute	Log-normal	Dropout threshold Number of clusters	0-1 (default: 0.5) Correct value given the simulation	( Li & Li, 2018)
DrImpute *	ZINB	Remaining zeros Number of clusters	0-1 (default: 0) Correct value given the simulation	( Gong *et al*., 2018)
SAVER	ZINB	Which genes to impute	Top 1%–100% most highly expressed (default: 100%)	( Huang *et al*., 2018)
MAGIC	NA	Diffusion time, K neighbours	1–8 (default: allow algorithm to choose) 5–100 (default: 12)	( van Dijk *et al*., 2018)
knn-smooth	NA	K neighbours	5–100 (default: number of cells / 20)	( Wagner *et al*., 2017)
dca	ZINB	Hidden layer size +5 others	2–64 (default: 32) Software defaults	( Eraslan *et al*., 2019)

*Note: All methods were applied to raw counts as intended by the authors and returned values on that scale, except for DrImpute which as per the documentation was applied to log2(CPM+1) and returned log-scaled values. CPM = counts per million.

### Negative binomial simulations

As an initial test of imputation methods and to understand the effect of various method-specific parameters on imputation we simulated data from a negative binomial model, which is known to be a good model of bulk and single-cell RNA-seq data ( Grün *et al*., 2014; Robinson & Smyth, 2007). Expression matrices containing 1000 cells, equally spread across two cell-types, and 500 genes, with mean expression ranging from 10 ^-3^-10 ^4^, were simulated. Half of the genes were differentially expressed (DE) by an order of magnitude between the two cell-types, half were drawn independently. Since there are no added dropouts in these simulations the desired behavior for model-based imputation methods is to leave the data as is. Whereas for data-smoothing the desired behaviour would be to assign non-DE genes a constant value across all cells. Ten such expression matrices were independently simulated. Each imputation method was run on each replicate with a range of parameter values ( Table 1). Significant gene-gene correlations were identified using Spearman correlation with a conservative Bonferroni multiple testing correction (q < 0.05) to avoid distributional assumptions on the imputed values. We specifically choose the Bonferroni correction to avoid issues arising from an abundance of very low p-values resulting from imputation of the strong DE genes present in these simulations. A distorted p-value distribution would be problematic as it violates the assumptions of the more typical false discovery rate correction ( Benjamini & Hochberg, 1995).

Correlations were calculated directly on the output of the imputation methods which was on the count-scale for all methods except DrImpute for which both the input and output are on a log-scale. However, since we used the non-parametric Spearman correlation the effect of different scales is minimal and largely restricted to distortions due to normalization biases and the addition of a pseudo-count. Correlations involving not DE genes or in the incorrect direction were considered false positives.

### Splatter simulations

Splatter ( Zappia *et al*., 2017) was used to generate 60 simulated single-cell RNASeq count matrices using different combinations of parameters ( Table 2). Each simulated dataset contained 1,000 cells split into 2–10 groups and 1,000–5,000 genes of which 1–30% were differentially expressed across the groups. For simplicity all groups were equally sized and were equally different from one another. Half the simulations assumed discrete differentiated groups, whereas the other half used the continuous differentiation path model. We also considered the effect of four different amounts of added dropouts plus the no-added dropout model. These simulation parameters broadly matched real scRNA-seq data, with lower dropout rates being more similar to 10X Chromium data and higher dropout rates being more similar to Smart-seq2 data ( Figure S1). Each simulated dataset was imputed with each method using default parameters.

**Table 2.** Splatter parameters.

nGenes *	%DE (total) *	Dropouts (midpoint) **	nGroups	Method	Seed
1000 2000 5000	1% 10% 30%	None (45%) 1 (70%) 2 (80%) 3 (88%) 4 (94%)	2 5 10	Groups	8298 2900

*Randomly selected for each possible combination of the other four parameters.

** Numbers in parentheses indicate proportion of the expression matrix that was “0” values.

Accuracy of each imputation method was evaluated by testing for differentially expressed (DE) genes between the groups used to simulate the data. To avoid issues of different imputation methods resulting in data best approximated by different probability distribution, we employed the non-parametric Kruskal-Wallis test ( Kruskal & Wallis, 1952) with a 5% FDR to identify significant DE genes. The Kruskal-Wallis test is the multi-group extension of the Mann-Whitney-U test that performs a single test per gene regardless of the number of groups to compare ensuring equivalent power and multiple-testing corrections across simulations. Since this test is relatively low-power it is likely to underestimate the number of DE genes compared to alternatives. To filter DE results by effect size, in addition to significance, the magnitude of the DE (i.e. effect size) was estimated as the maximum log2-fold-change across all pairs of clusters. Only genes where the magnitude of the DE exceeded a specified threshold and were significant after a 5% FDR were called as DE in this case.

### Permuted Tabula Muris datasets

Six 10X Chromium and 12 Smart-seq2 datasets were chosen from the Tabula Muris (8) consortium data such that: i) there were at least two cell types containing >5% of the total cells and ii) there were between 500–5,000 cells after filtering ( Table S1). Each dataset was preprocessed to remove cell-types accounting for <5% of total cells, and any cells not assigned to a named cell-type. Genes were filtered to remove those detected in fewer than 5% of cells.

We selected the two most similar cell-types in each dataset using the Euclidean distance between their mean expression profiles. Differential expression of each gene between these cell-types was evaluated using a Mann-Whitney-U test, which is the two-sample equivalent of the Kruskal-Wallis test, on the log2 library size normalized counts (pseudo-count of 1). Genes with a raw p-value > 0.2 were then permuted across the selected cell-types to eliminate any residual biological signals. Permuted raw counts were obtained by de-logging and de-normalizing the permuted log2-normalized expression to avoid library-size confounders.

Each imputation method was applied to the full dataset after permutation using default parameters ( Table 1). False-positives introduced by each imputation was assessed by applying the Mann-Whitney-U test to test for differential expression between the two chosen cell-types. A Bonferroni multiple-testing correction was applied to ensure a consistent level of expected total false positives of less than 1.

### Reproducibility of markers

We utilized the six tissues for which there exists matching Smart-seq2 and 10X Chromium data from the Tabula Muris (8) to evaluate the reproducibility of imputation results. These datasets were filtered as described above, and any cell-types not present in both pairs of the matching datasets were excluded. Each imputation method was applied to the datasets without any permutation.

Marker genes were identified in each imputed dataset using a Mann-Whitney-U test, which is the two-sample version of the Kruskal-Wallis test, to compare each cell-type against all others, and effect size was calculated as the area under the ROC curve for predicting each cell-type from the others ( Kiselev *et al*., 2017). Genes were assigned to the cell-type for which they had the highest AUC. Significant marker genes were defined for each imputed dataset using a 5% FDR and an AUC over a particular threshold. Reproducibility was evaluated by determining the number of genes that were significant markers in both of a matching pair of datasets and were markers of the same cell-type. We used marker genes rather than DE genes to simplify the evaluation of reproducibility, since each gene was assigned to a single cell-type per dataset rather than a matrix of fold-changes across all pairs of cell-types. In addition, since these datasets contained clearly distinct cell-types nearly all genes were differentially expressed between some pairs of cell-types (e.g. B-cells and lung stromal cells). The presence of such outliers could potentially distort overall DE reproducibility measures.

## Results

**Figure 1.**
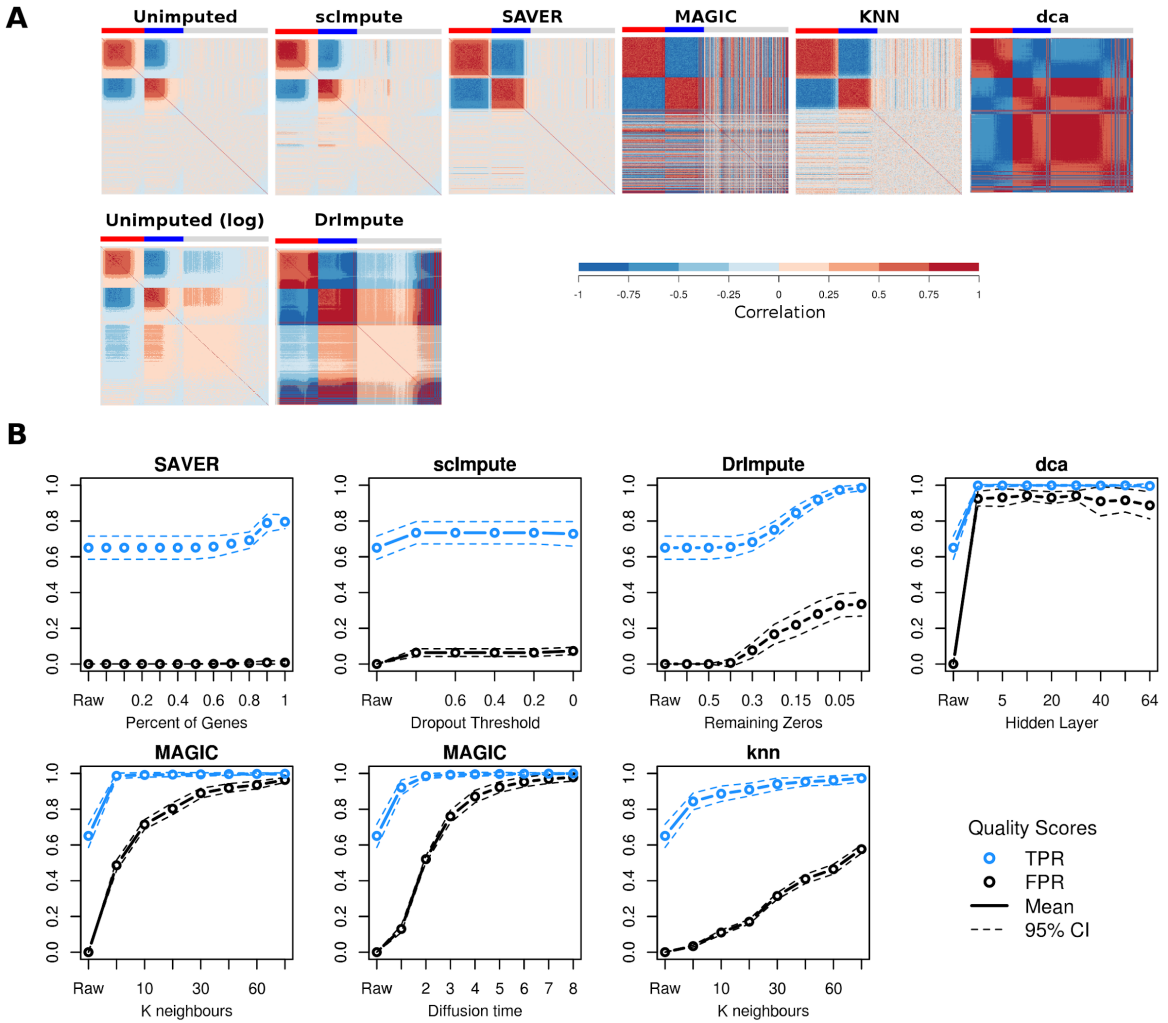
False gene-gene correlations induced by single-cell imputation methods. ( **A**) Gene-gene correlations before and after imputation using suggested parameter values: SAVER (all genes), MAGIC ( *k*=12, *t*=3), knn ( *k*=50), scImpute (threshold=0.5), DrImpute (remaining zeros=0), dca (hidden layer size=32). Coloured bars indicate genes highly expressed (red) or lowly expressed (blue) in one cell population vs the other, or genes not differentially expressed between the populations (grey). Genes are ordered left to right by DE direction then by expression level (high to low). ( **B**) False positive and true positive gene-gene correlations (p < 0.05 Bonferroni multiple testing correction) as imputation parameters are changed. “Raw” indicates results for unimputed data. Dashed lines are 95% CIs based on 10 replicates.

We tested three published imputation methods, SAVER ( Huang *et al*., 2018), scImpute ( Li & Li, 2018) and DrImpute ( Gong *et al*., 2018), two data-smoothing methods MAGIC ( van Dijk *et al*., 2018) and knn-smooth ( Wagner *et al*., 2017) and one autoencoder-based method dca ( Eraslan *et al*., 2019). We applied each method with the default parameter values ( Table 1) to data simulated from a simple negative binomial, since technical noise in scRNA-seq data has been observed to follow a negative binomial distribution since technical noise in scRNA-seq data has been observed to follow a negative binomial distribution ( Grün *et al*., 2014). All the imputation methods increased the sensitivity to detect gene-gene correlations between the lowly expressed DE genes. However, only SAVER strengthened the correlations between lowly expressed DE genes without generating false positive gene-gene correlations between independently drawn genes ( Figure 1A). Since SAVER models expression data using a negative binomial, it is expected to perform well on this simulated data. MAGIC and dca generated very strong false positive correlations ( *r* > 0.75) at all expression levels, whereas DrImpute, which only imputes zero values, created false positive correlations mostly among lowly expressed genes. Knn-smooth and scImpute produced a few false-positive correlations among moderately-expressed genes using default parameters.

Choice of parameter values has a large influence imputation results ( Figure 1B). Five of the imputation methods required the user to set at least one parameter *a priori*, only SAVER did not. We varied the thresholds scImpute and DrImpute use to determine which zeros to impute. For scImpute some of the lower and moderate expression values were imputed even at a very strict probability threshold ( *p* > 0.8), but changing the threshold had little effect on the imputation. As expected for DrImpute, imputing a greater proportion of zeros generated more false positive gene-gene correlations. Knn-smooth and MAGIC both perform data smoothing using a k-nearest-neighbour graphs between cells. Increasing the number of nearest-neighbours ( *k*) produces smoother data and more false-positive correlations ( Figure 1B). MAGIC provides a default value for *k* but no indication of how this parameter should be adjusted for different sized datasets, whereas knn-smooth provided no default value but a rough suggestion to scale the value depending on the total number of cells. MAGIC also utilizes a second parameter, time ( *t*), for the diffusion process acting on the graph which by default is algorithmically estimated for the dataset. Longer diffusion times produce smoother data and more false positives. Autoencoders involve a large number of parameters and these can have a large effect on performance ( Hu & Greene, 2018). For simplicity, we only considered the size of the hidden layer in this study. A larger hidden layer slightly reduced the tendency to generate false-positive gene-gene correlations.

These simple simulations contained only two cell-types and no technical confounders such as library-size or inflated dropout rates that are observed in some scRNA-seq datasets. For a more comprehensive evaluation of imputation methods we simulated data using Splatter ( Zappia *et al*., 2017). We simulated data with 1,000 cells split into 2–10 groups and 1,000–5,000 genes of which 1–30% were differentially expressed across the groups. We considered four different levels of zero inflation and no zero inflation ( Table 2). Each simulated dataset was imputed with each method using the default parameters ( Table 1). To score each imputation we considered the accuracy of identifying differentially expressed genes between the groups using the non-parametric Kruskal-Wallis test ( Kruskal & Wallis, 1952).

**Figure 2.**
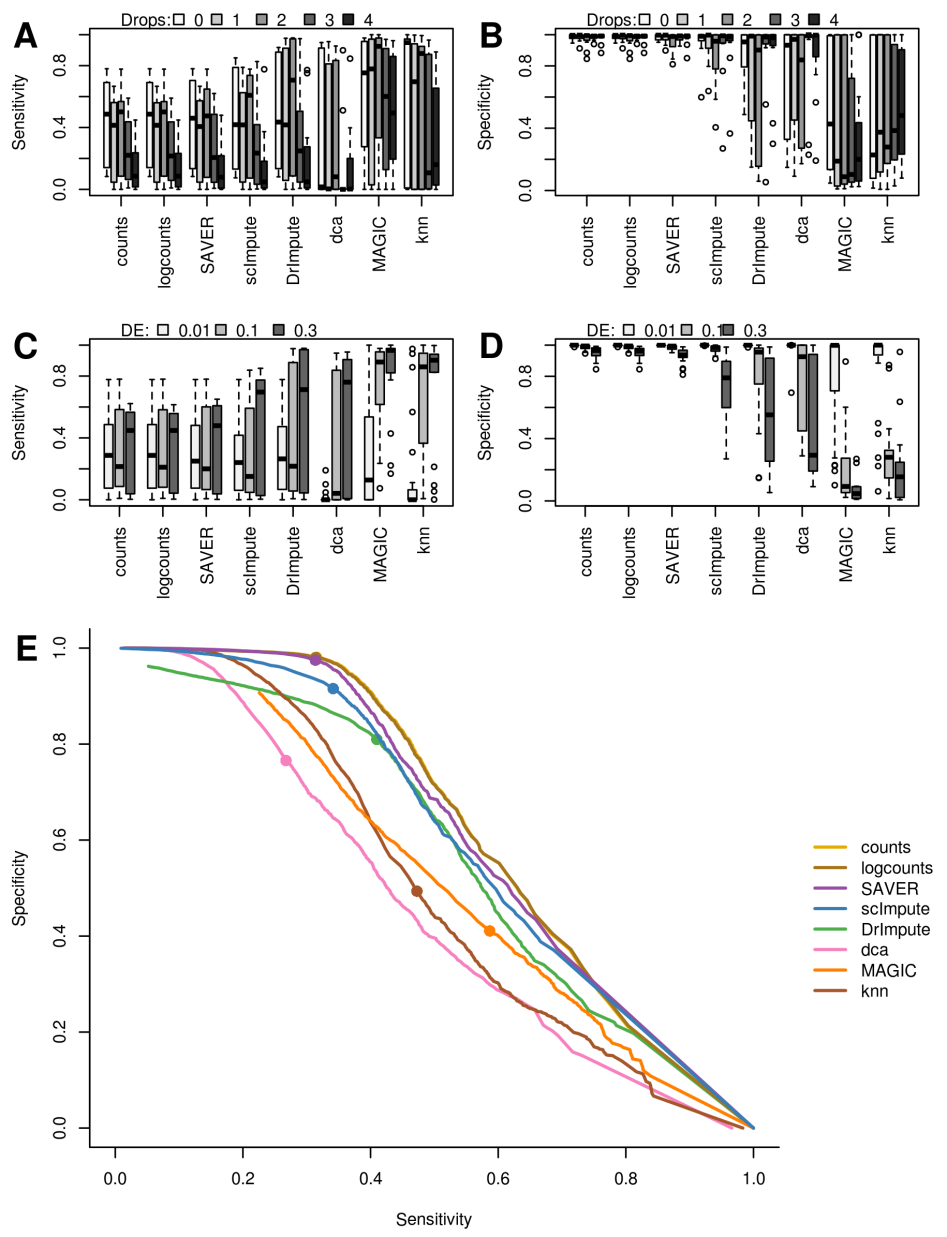
Accuracy of detecting differentially expressed (DE) genes in splatter simulations before and after imputation with each method. ( **A** & **B**) Zero inflation decreases sensitivity of DE which most imputation methods fail to correct. ( **C** & **D**) Strong true signals (high proportion of DE genes) decreases specificity particularly for data-smoothing methods. ( **E**) Average ROC curves across all simulations, solid dots indicate 5% FDR. Counts were normalized by total library size prior to testing DE, and “logcounts” are log2(normalized counts+1).

None of the imputation methods significantly outperformed the others or the unimputed data based on the sensitivity and specificity. While both knn-smooth and MAGIC have increased sensitivity, they have very low specificity, whereas SAVER and scImpute are very similar to the unimputed data with high specificity but relatively low sensitivity ( Figure 2A & B). DrImpute and dca were in between the two extremes with somewhat higher sensitivity and lower specificity than SAVER and scImpute. Both scImpute and DrImpute are designed specifically to only impute excess zeros but neither showed a clear improvement over the raw counts when the simulations contained various levels of zero inflation ( Figure 2A & B). By contrast, both smoothing methods, MAGIC and knn-smooth, retained relatively high sensitivity even at high dropout-rates, albeit with low specificity.

All methods except SAVER readily introduced false-positive differential expression, as demonstrated by a drop in specificity, when 30% of genes were DE ( Figure 2D). We also observe a significant but smaller drop in specificity for the normalized but unimputed data. We hypothesize that slight biases when correcting for library-size in the presence of strong biological differences may be amplified by the imputation methods. Biases due to counts-per-million library-size normalization, in the presence of strong DE are a known issue from bulk RNASeq analysis ( Bullard *et al*., 2010). Both MAGIC and knn-smooth automatically use counts-per-million to normalize data before smoothing, and dca using log-transformed data to estimate library-size in its model which explains why it displays similar bias to DrImpute, which imputes log2-normalized data ( Figure 1A). 

 Importantly, when the trade-off between sensitivity and specificity was considered across significance thresholds we found that imputation methods generally performed worse than the raw data ( Figure 2E). This indicates that similar sensitivities to those observed in imputed data could be achieved with a higher specificity by simply lowering the significance threshold for the DE test. The only exception is SAVER which performed almost identically to the unimputed data. Overall, model-based methods performed better than smoothing-methods when both sensitivity and specificity are taken into account.

**Figure 3.**
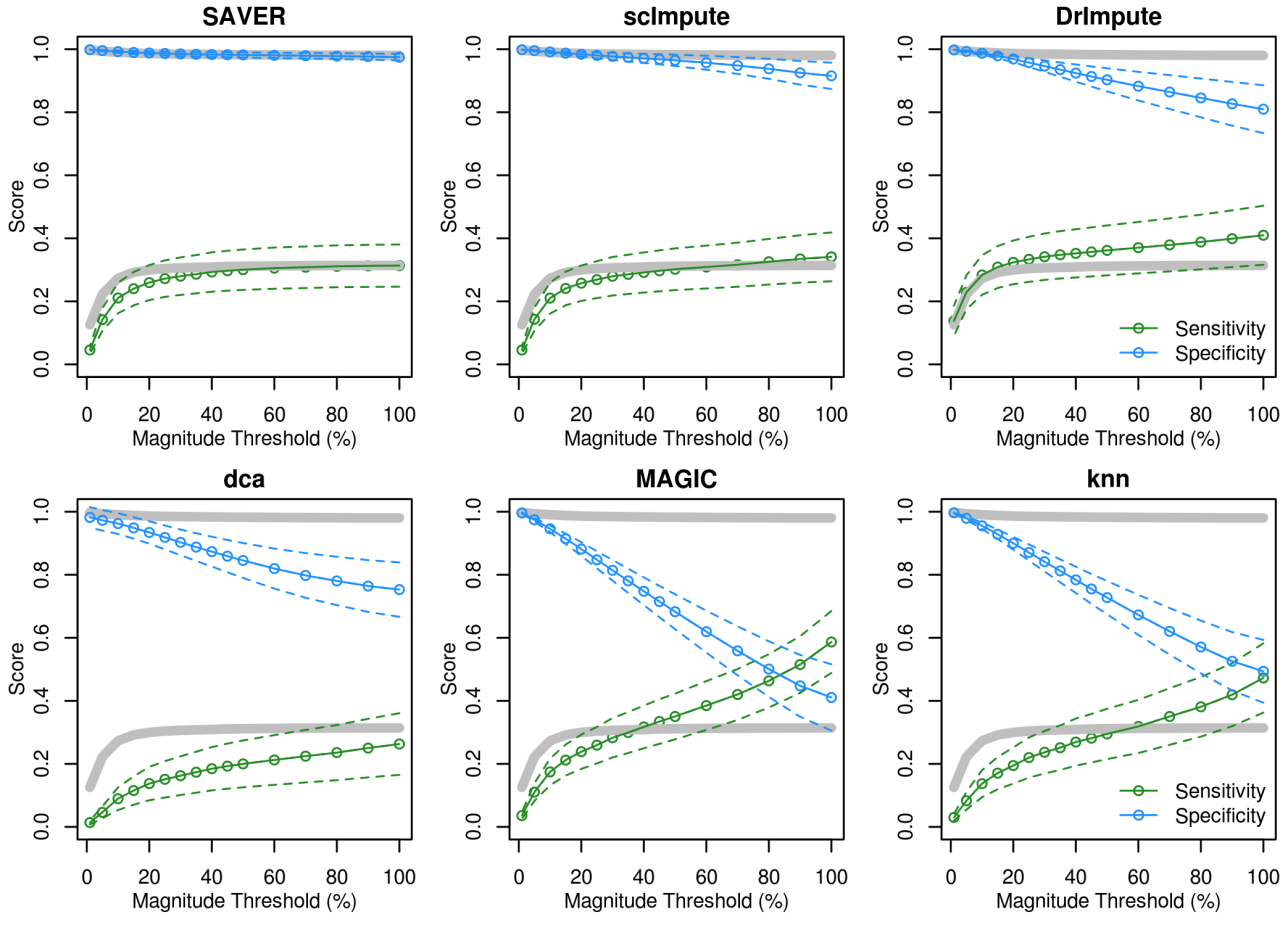
Filtering by the magnitude of expression differences restores specificity in imputed data. Sensitivity (green) and specificity (blue) of each imputation method applied to the splatter-simulated data, when restricting to only the top X% of genes by fold-change. Dashed lines indicate 95% CI. Grey lines indicate results for the un-imputed data.

It is possible that the bulk of false-positives generated by imputation methods result from small biases or sampling noise being amplified to reach statistical significance. If this is true, then filtering DE genes by magnitude in addition to significance should restore the specificity of such tests on imputed data. We observed this to be the case when an additional threshold was set based on the Xth percentile highest log2 fold-change across the whole dataset ( Figure 3). However, sensitivity also declined as the fold-change threshold was made more stringent. Again, we observe that data-smoothing offers a worse trade-off between sensitivity and specificity than the un-imputed data, whereas model-based imputation is very close to the un-imputed data.

**Figure 4.**
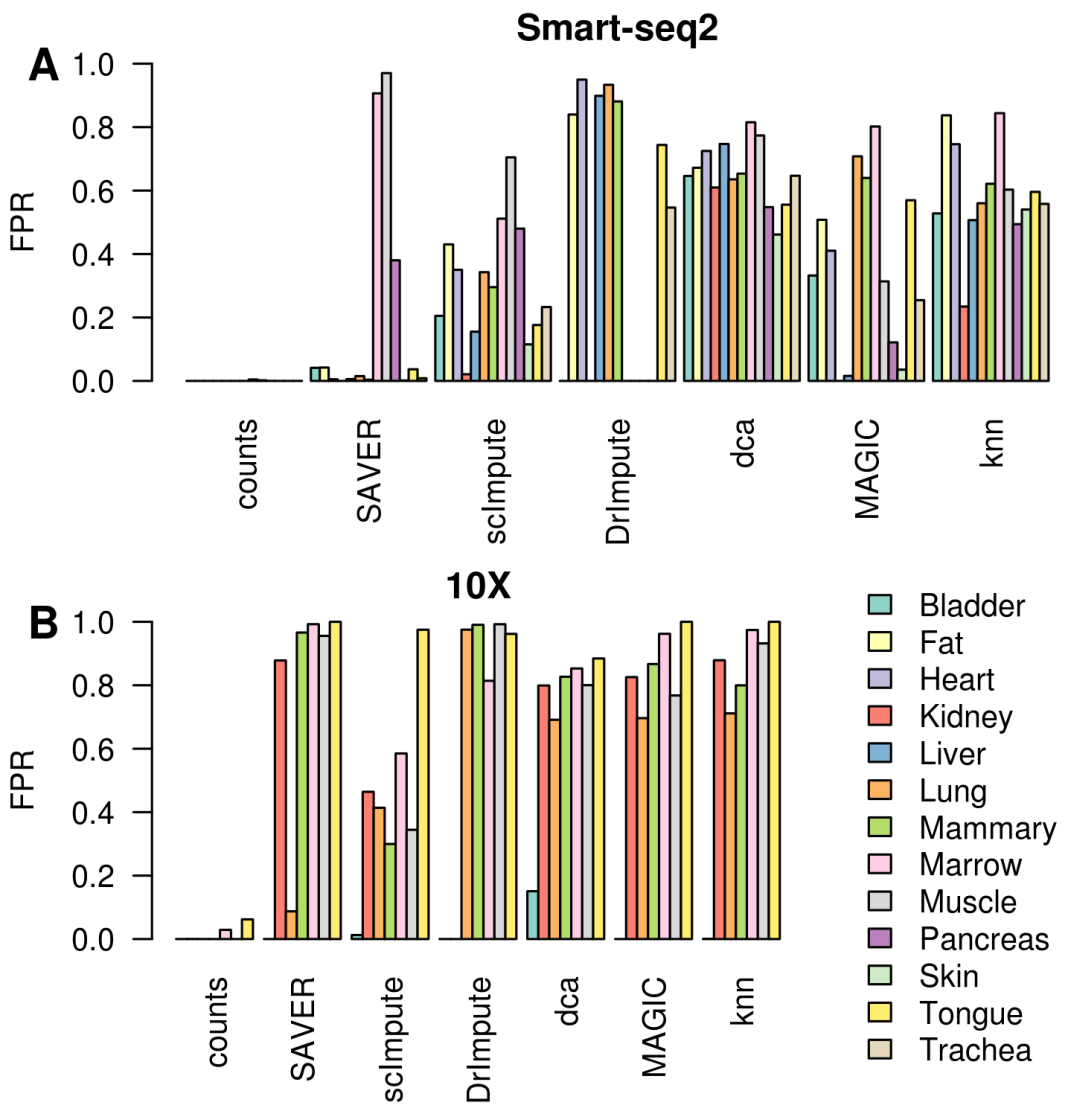
High variability in false positives induced by imputation across datasets regardless of sequencing technology. ( **A**) Smart-seq2 datasets, ( **B**) 10X Chromium datasets. Non-differentially expressed genes were permuted prior to imputation.

Splatter is a widely used simulation framework for scRNA-seq but may not fully capture the complexities of real scRNA-seq data. To test the performance of each imputation method on real scRNA-seq data we selected 12 tissues from the Tabula Muris database ( Tabula Muris Consortium *et al*., 2018) and applied the imputation methods to the Smart-seq2 and 10X data separately. Since the ground truth is not known for these data, we selected two cell-types from each dataset and permuted the expression of those genes that were not differentially expressed between them (p > 0.2) to generate a set of genes that we could confidently consider as being not differentially expressed (Methods). Using these as ground truth we could estimate the number of false positive differentially expressed genes introduced by each imputation method. Strikingly, we observed a very high variability between datasets which appears to be unrelated to the experimental platform ( Figure 4A & B). MAGIC, dca and knn-smooth consistently produced large numbers of false positives (20–80%). Whereas, DrImpute and SAVER were extremely variable producing few to no false positives in some datasets and over 90% false positives in others.

Imputation methods generated more false-positives in the sparser 10X Chromium data than on the higher depth Smart-seq2 data. This was not due to genes failing to conform to the negative binomial distribution ( Figure S2). Rather, it is likely due to relatively stronger real signals and greater power in the large 10X datasets as seen in our splatter simulations ( Figure 2C & D), or due to biases in library size correction. We found that dca, MAGIC, knn-smooth, SAVER and scImpute tend to bias all the permuted genes in the same direction ( Figure S3), though interestingly the direction of the bias depends on the method with MAGIC and SAVER biased in one direction and knn-smooth and scImpute biased in the opposite direction. Dca was less consistent, sometimes being more similar to MAGIC and sometimes more similar to knn-smooth. This suggests an error in library-size correction is responsible for their poor performance on some datasets. In contrast, DrImpute imputed genes in random directions suggesting it is amplifying random noise in the dataset.

**Figure 5.**
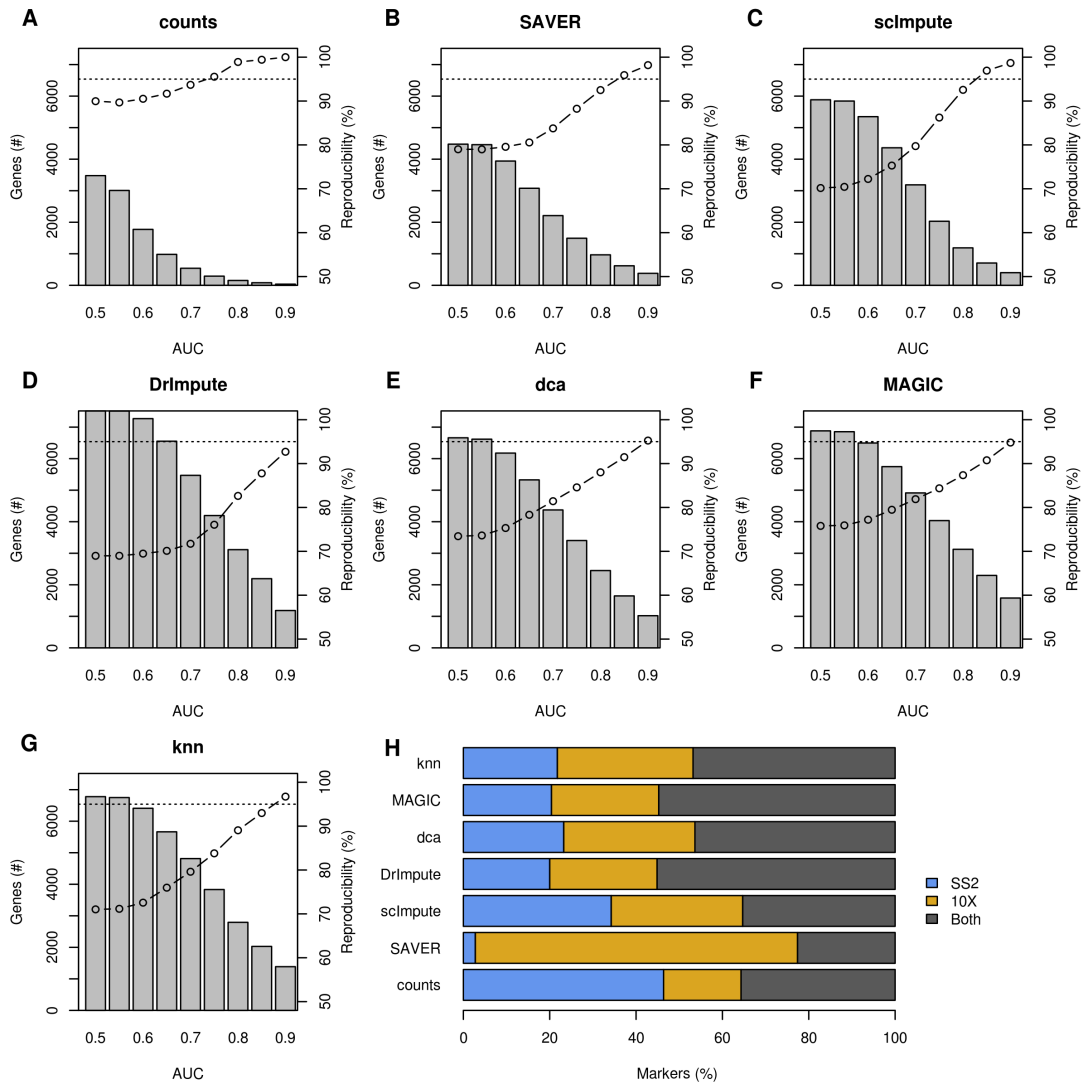
Reproducibility of marker genes can be restored in imputed data using a strict effect-size threshold. ( **A**– **G**) Markers were identified in 10X Chromium and Smart-seq2 data for six different mouse tissues. The average number of markers (bars, left axis) and proportion reproducible across both datasets (line, right axis) are plotted. Only significant markers (5% FDR) exceeding the AUC threshold were considered. ( **H**) Proportion of markers that were unique to the Smart-seq2 (blue, SS2), or 10X Chromium (yellow), or both (dark grey).

To complement the false positives in the permuted data, we used a marker being associated with the same cell-type in both 10X and Smart-seq2 data as evidence that a gene is a “true” marker. This was a necessary but flawed assumption, since the complete list of true markers is not known. Systematic differences in cell-size, and hence gene-detection rates, may result in reproducible biases in imputation across multiple datasets. In addition, even if markers are randomly associated to cell-types, a portion will agree just by chance. Thus, the proportion of irreproducible markers should be considered an underestimation of the true number of erroneous markers. We identified marker genes using a Mann-Whitney-U test, comparing one cell-type to the others in that tissue. Markers were selected by significance (5% FDR) and magnitude (AUC > *T*). Each marker was assigned to the cell-type for which it had the highest AUC. To prevent differences in power from affecting the results, reproducibility was measured as the fraction of those markers that were significant in both dataset that were also markers for the same cell-type ( Figure 5). 

All of the imputation methods increased the absolute number of reproducible significant markers ( Figure S4). However, these were mixed in with a larger number of irreproducible markers ( Figure 5). Without imputation, 95% of genes that were significant markers in both datasets were highly expressed in the same cell-type. After imputation, this dropped considerably depending on the AUC threshold. Decreasing the magnitude threshold led to more markers assigned to contradictory cell-types in the imputed Smart-seq2 and 10X Chromium datasets. Unimputed data retained >90% concordance in cell-type assignments of significant markers regardless of the AUC threshold, this fell to 70–80% in imputed data when a low AUC threshold is used. However, employing an AUC threshold of 0.9 increased reproducibility in imputed data back to 95% while retaining more markers than in the un-imputed data. When we considered the overall concordance of the marker test results across dataset, we found that the un-imputed data had the highest concordance in every tissue ( Figure S5).

When comparing across imputation methods applied to the same Tabula Muris dataset, we found variable concordance between methods ( Figure S6). Overall 5–35% of markers were assigned to different cell-types depending on the imputation method(s) used. As we observed for the permuted genes, imputation methods tended to two different groups depending on their particular bias, one containing MAGIC, SAVER and dca, the other containing scImpute, DrImpute and knn-smooth. This discrepancy is concerning, since it could cause the biological interpretation of a dataset to depend on the choice of imputation method.

**Figure 6.**
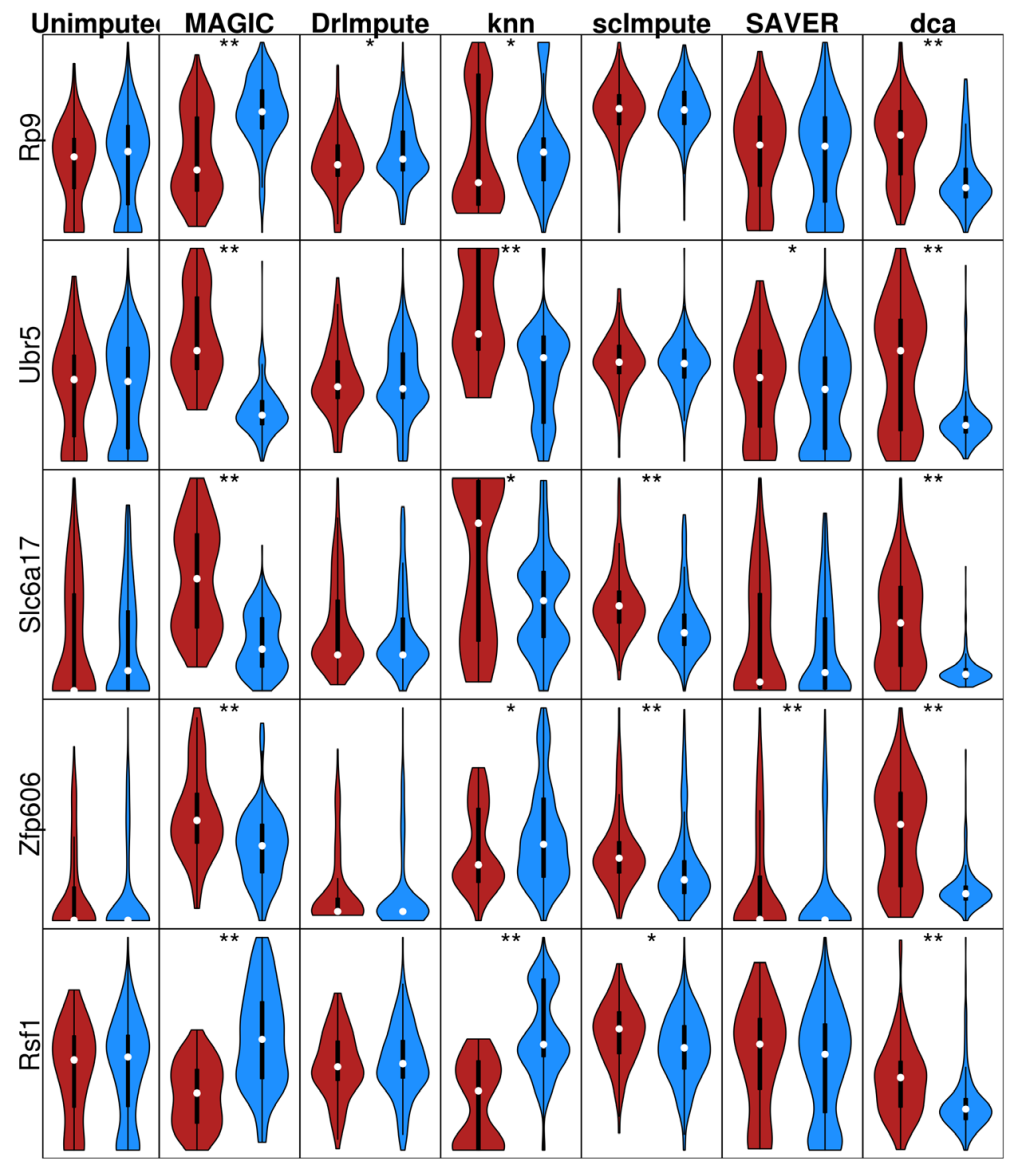
Examples of false positive DE induced by imputation of Pancreas Smart-seq2 data. Unimputed indicates the permuted normalized log-transformed expression. Red = PP cell, Blue = A cell. * = p < 0.05, ** = significant after Bonferroni (q < 0.05) correction.

Inspection of the false positives generated by imputation of the permuted real data revealed method-specific distortions of the gene expression values ( Figure 6). SAVER had little effect on the distribution shape, but did eliminate zeros from the data. scImpute and DrImpute both tended to make the distribution more gaussian. In contrast, MAGIC and knn-smooth tended to generate bimodal expression distributions. The tendency towards bimodality could be problematic for downstream analysis since many methods, e.g. PCA and differential expression, assume either negative binomial or gaussian distributions. Many of these genes were differentially expressed after imputation, despite being permuted previously. Interestingly, the direction of differential expression was not always consistent across imputation methods, for instance *Zfp606* was more highly expressed in PP cells than A cells after imputation using MAGIC but the inverse was true after imputing with knn-smooth.

## Discussion

We have shown that imputation for scRNA-seq data may introduce false-positive results when no signal is present. On simulated data all the methods except SAVER generated some degree of false positives ( Figure 1 & Figure 2). We find the fundamental trade-off between sensitivity and specificity, inherent to their definition, cannot be overcome with imputation ( Figure 2 & Figure 3). On permuted real data, imputation results were more variable ( Figure 4), and even SAVER generated large numbers of false positives in some datasets. Considering a scenario where a signal is present, we found that imputation also reduced the reproducibility of marker genes, unless strict magnitude thresholds were imposed ( Figure 4 & Figure 5). In addition to false-positives, distortions in expression distributions ( Figure 6) may cause imputed data to violate assumptions of some statistical tests.

We found that different imputation methods favour either sensitivity or specificity but that none of them result in an overall improvement for detecting differential expression ( Figure 2). MAGIC, dca and knn-smooth which are data-smoothing methods, as such they adjust all expression values not just zeros. Since they impose larger alterations on the data, these methods generate many more false positives than methods which only impute zero values. They also have a greater sensitivity, although a similar sensitivity could be achieved by simply reducing stringency of the significance test which would generate fewer false positives. In contrast, model-based methods which only impute low expression values, generated fewer false positives but had minimal improvements to sensitivity. Adding an effect size threshold can reduce false positives generated by imputation and shift the trade off back to lower sensitivity but higher specificity ( Figure 3, Figure 5).

These trade-offs reflect the fundamental limitation of current approaches to single-cell RNASeq imputation, namely that the methods considered here only use the information present in the original data. Hence no new information is gained, making it analogous to simply lowering the significance threshold of any statistical test applied to the data ( Fawcett, 2006). However, as large reference datasets such as the Human Cell Atlas ( Regev *et al*., 2017; Rozenblatt-Rosen *et al*., 2017), and equivalent projects in other species ( Han *et al*., 2018; Plass *et al*., 2018; Tabula Muris Consortium *et al*., 2018; Zeisel *et al*., 2018) are completed it will be possible to employ methods which borrow information from them for imputation such as the recently released SAVER-X method ( Wang *et al*., 2018). However, reference-based imputation is limited by the completeness of the external dataset. Alternatively, models could be developed to use gene-gene correlations derived from large external databases of expression data ( Obayashi *et al*., 2008), while more generalizable such methods may not capture cell-type specific relationships.

In our simulations, we have employed the conservative Bonferroni correction and we have ignored potential confounders, such as batch-effects, that imputation methods could mistake for the true structure. Thus, the false-positive rates shown here should be considered underestimates of the true false-positive rates. Similarly, technical confounders and random chance will generate some degree of agreement between markers found in two dataset, which we did not account for in the analysis of Smart-seq2 and 10X Chromium datasets, which again results in underestimating the false-positive rates in imputed data. False-positives resulting from imputation may be much higher than those observed here in the worse case scenario of strong batch-effects, differing cell-size within a sample, and confounding variability such as stress response. Since imputation will amplify any and all possible signals, including random noise, we expect confounding signals to be amplified as well.

We have shown that the circularity induced by imputation causes the outputs of imputation methods to violate the assumptions of statistical tests commonly applied to single-cell RNA-seq. This inflates the number of false-positive gene-gene correlations, cell-type markers, and differentially expressed genes. In general, our results suggest that it is better to decrease the significance threshold applied to the test than to apply an imputation method to increase sensitivity in sparse datasets. However, imputation may still be useful for visualization of single-cell RNA-seq data since it exaggerates existing structure within the data. Of the methods we tested, SAVER was the least likely to generate false positives, but its performance was variable when tested on real data.

If imputation is used, combining SAVER with an effect size threshold is the best option to avoid irreproducible results. Alternatively, verifying the reproducibility of results across multiple datasets or multiple imputation methods can eliminate some false positives. However, our results highlight that statistical tests applied to imputed data should be treated with care. Moreover, as our study only focused on the expression levels, we cannot exclude the possibility that imputation will be beneficial when considering other aspects, e.g. clustering or pseudotime alignment. Although a previous benchmarking study showed good results for positive controls, our study highlights the importance of considering negative controls when evaluating imputation methods.

## Data and software availability

***Tabula Muris data***

Smart-seq2 https://doi.org/10.6084/m9.figshare.5715040.v1 ( Consortium, The Tabula Muris, 2017a).

10X Chromium https://doi.org/10.6084/m9.figshare.5715040.v1 ( Consortium, The Tabula Muris, 2017b).

***R packages***

MAGIC: Rmagic (v0.1.0) https://github.com/KrishnaswamyLab/MAGIC

DrImpute: DrImpute (v1.0) https://github.com/ikwak2/DrImpute

scImpute: scImpute(v0.0.8) https://github.com/Vivianstats/scImpute

SAVER: SAVER(v1.0.0) https://github.com/mohuangx/SAVER

Knn-smooth: knn_smooth.R (Version 2) https://github.com/yanailab/knn-smoothing

Scater: scater(v1.6.3) : https://www.bioconductor.org/packages/release/bioc/html/scater.html

Splatter: splatter(v1.2.2) : https://bioconductor.org/packages/release/bioc/html/splatter.html

Permute: permute(v0.9-4) : https://cran.r-project.org/web/packages/permute/index.html

***Python/anaconda packages:***

Dca : dca(v0.2.2): https://github.com/theislab/dca

***Custom scripts:*** https://github.com/tallulandrews/F1000Imputation

## Supplementary material

Supplementary File 1: File containing Table S1 (Tabula Muris permuted datasets) and Figure S1 (Comparison of splatter simulations to real scRNA-seq data), Figure S2 (Distribution fits of false-positives among permuted genes), Figure S3 (Heatmap visualization of false-positives among permuted genes), Figure S4 (Absolute number of reproducible markers after imputation), Figure S5 (Correlation between marker significant tests across 10X and Smart-seq2), Figure S6 (Proportion of markers that are assigned to different cell-types by different imputation methods).
