# Supplementary material for: False signals induced by single-cell imputation
Source: F1000Res. 2019 Mar 5;7:1740. Originally published 2018 Nov 2. [Version 2] doi: 10.12688/f1000research.16613.2 (PMC6415334; doi:10.12688/f1000research.16613.2)
Supplement: Supplementary file 1 [file f1000research-7-20056-s0000.tgz › c59ce0d7-ff96-4680-90cc-436f71bdae47_Supplemeantary_File_1._ImputationSupplementary.pdf]

Supplementary

Table S1 : Tabula Muris permuted datasets.

Tissue	Technolog y	Cells (filtered)	Closest cell-types	Permuted Genes	Best Fit Model*
Heart	Smartseq2	4098	Smooth muscle cell<br>Endothelial cell	2912	ZINB: 99%<br>ZILN: 0%
Kidney	Smartseq2	517	Fenestrated cell<br>Endothelial cell	5728	ZINB: 77%<br>ZILN: 22%
Pancreas	Smartseq2	1204	Pancreatic PP cell<br>Pancreatic A cell	7300	ZINB: 95%<br>ZILN: 0%
Trachea	Smartseq2	813	Leukocyte<br>Epithelial cell	4156	ZINB: 97%<br>ZILN: 3%
Lung	Smartseq2	1198	Stromal cell<br>Endothelial cell	1862	ZINB: 91%<br>ZILN: 1%
Bladder	Smartseq2	1287	Bladder cell<br>Basal cell of urothelium	5460	ZINB: 97%<br>ZILN: 1%
Muscle	Smartseq2	1831	Skeletal muscle stem cell<br>Skeletal muscle cell	2665	ZINB: 99%<br>ZILN: 0%
Tongue	Smartseq2	1394	Keratinocyte<br>Basal cell of epidermis	3600	ZINB: 65%<br>ZILN: 0%
Liver	Smartseq2	646	Kupffer cell<br>Endothelial cell hepatic sinusoid	5288	ZINB: 80%<br>ZILN: 20%
Mammary	Smartseq2	2255	Basal cell<br>Luminal epithelial cell	2335	ZINB: 70%<br>ZILN: 0%
Marrow	Smartseq2	4442	Neutrophil<br>Granulocyte	1359	ZINB: 97%<br>ZILN: 0%
Skin	Smartseq2	2218	Epidermal cell<br>Basal cell of epidermis	4417	ZINB: 95%<br>ZILN: 1%
Mammary	10X	4295	T cell<br>B cell	4295	ZINB: 99%<br>ZILN:0%

Bladder	10X	2375	Bladder cell<br>Basal cell of urothelium	6239	ZINB: 90%<br>ZILN:0%
Muscle	10X	4224	T cell<br>B cell	3260	ZINB: 100%<br>ZILN: 0%
Marrow	10X	3285	Hematopoietic stem cell<br>B cell	2243	ZINB: 81%<br>ZILN: 0%
Kidney	10X	2447	Macrophage<br>Fenestrated cell	3630	ZINB: 99%<br>ZILN: 0%
Lung	10X	4247	Stromal cell<br>Endothelial cell	1936	ZINB: 90%<br>ZILN: 0%
Tongue	10X	7501	Keratinocyte<br>Basal epidermal cell	839	ZINB:43%<br>ZILN: 0%

*Proportion of permuted (not DE) genes that were better fit by either a zero-inflated negative binomial distribution (ZINB), or a zero-inflated log-normal distribution (ZILN). Missing percentages are genes that didn't fit either distribution.

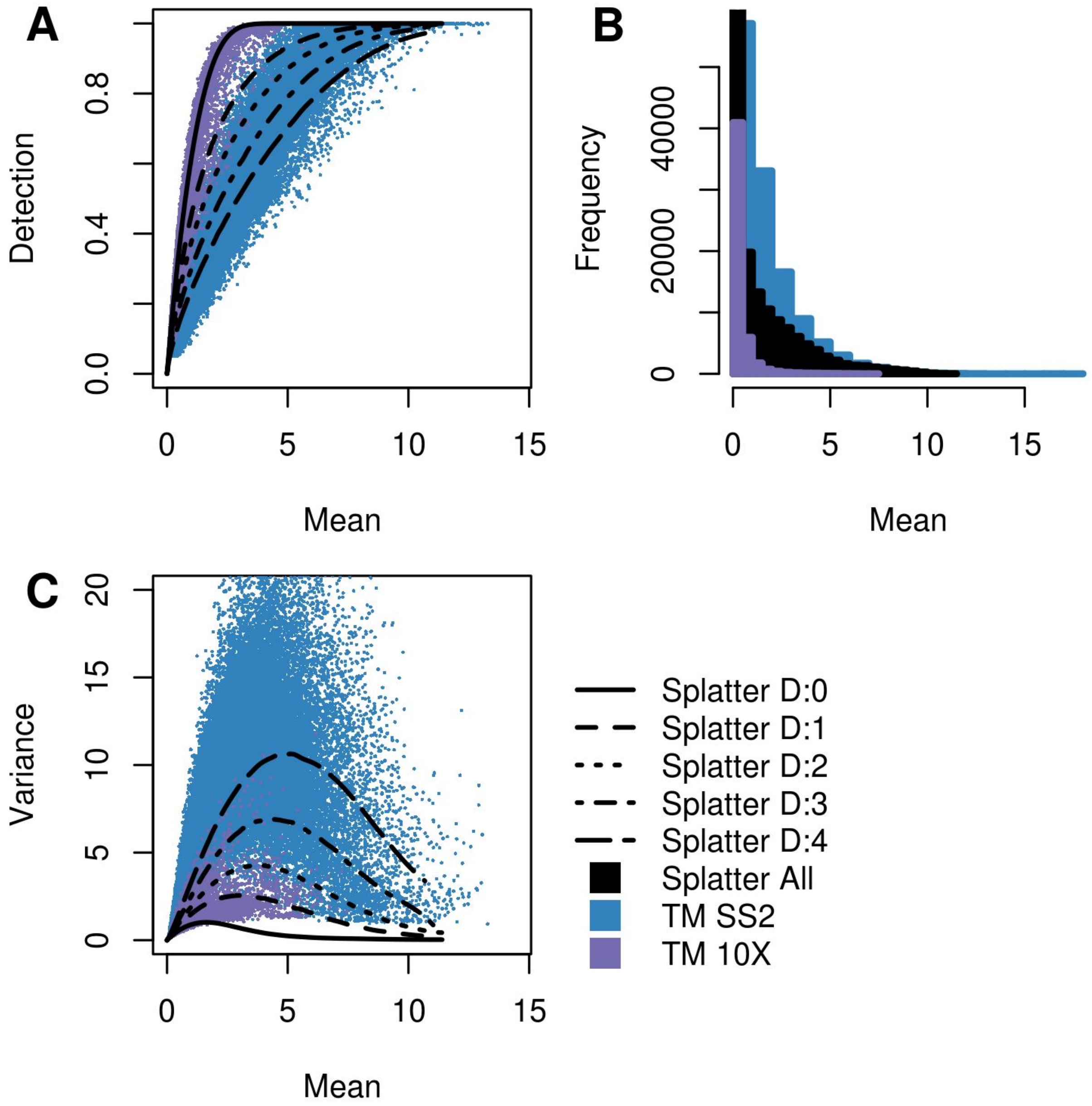

Figure S1 Comparison of Splatter simulations and real scRNA-seq data. Smartseq2 (blue) and 10X Chromium (purple) scRNA-seq data from the Tabula Muris dataset. Both were normalized and log2 transformed using scater. Results for splatter datasets with different values for the dropout-rate parameter: D, were spline-smoothed to generate curves. (A) Detection rate vs mean expression of genes across all cell-types in each tissue. (B) Distributions of mean expression levels. (C) Variance vs mean expression of genes.

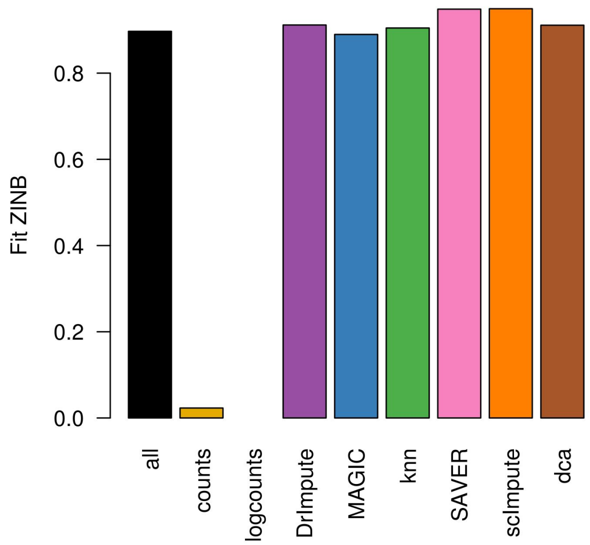

Figure S2 : **Imputed false differential expression is not due to genes violating a negative binomial.** Proportion of false-positive Tabula Muris markers that fit a zero-inflated negative binomial (ZINB) distribution better than a zero-inflated log-normal (ZILN) distribution. “All” indicates the proportion of all genes that were fit better by the ZINB distribution.

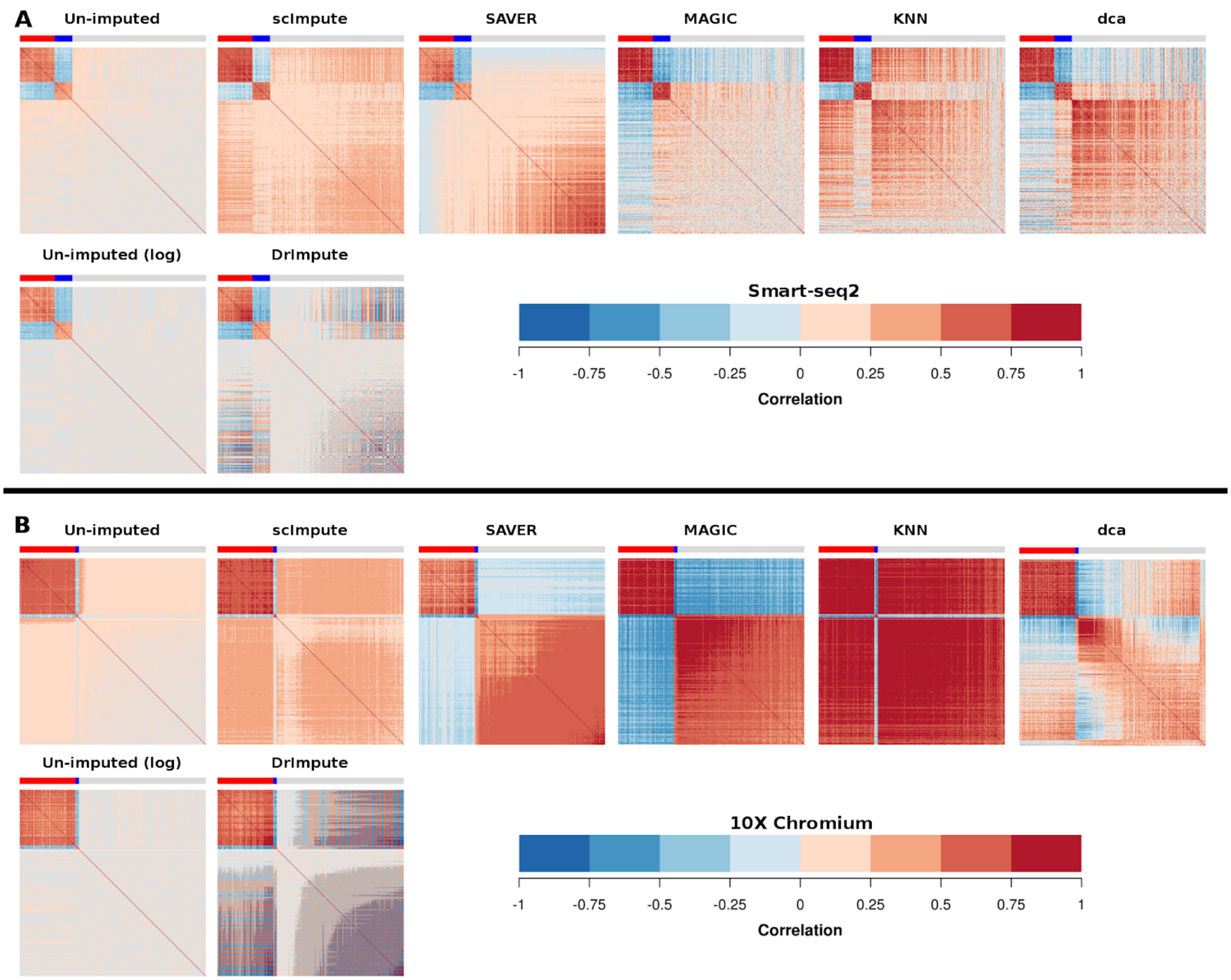

Figure S3: False gene-gene correlations induced by single-cell imputation methods. Gene-gene Spearman correlations between genes in permuted mouse tongue before and after imputation using suggested parameter values. Coloured bars indicate marker genes (AUC > 0.75, FDR 5%) for the two different cell-types (red & blue) or permuted genes (grey). Genes are ordered left to right by DE direction then by expression level (high to low). For visualization, 500 genes were randomly subset from Smart-seq2 (A) and 10X Chromium (B) ensuring at least 50% were the permuted genes.

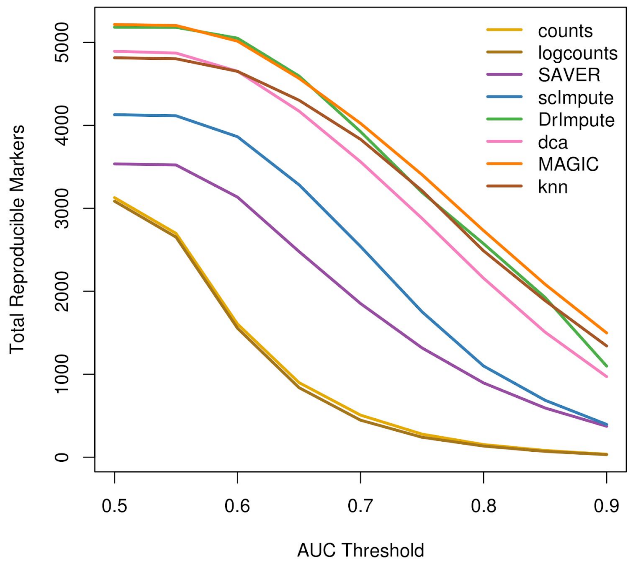

Figure S4: Absolute number of reproducible markers before and after imputation averaged across all Tabula Muris datasets. Markers were identified using a Mann-Whitney-U test, FDR 5%, and requiring an AUC score for classifying cells into the respective cell-type above a particular threshold. Genes identified as markers of the same cell-type in both Smart-seq2 and 10X datasets were considered reproducible.

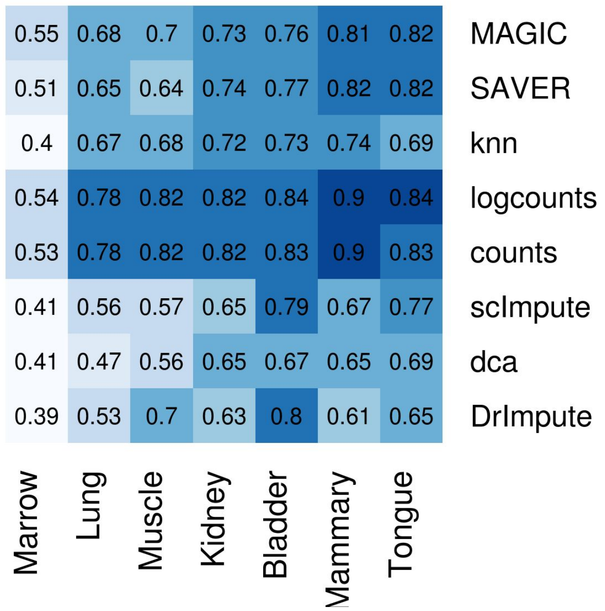

Figure S5: Concordance of markers test results across Tabula Muris datasets. Spearman correlations between marker p-values in matching 10X Chromium and Smart-seq2 datasets.

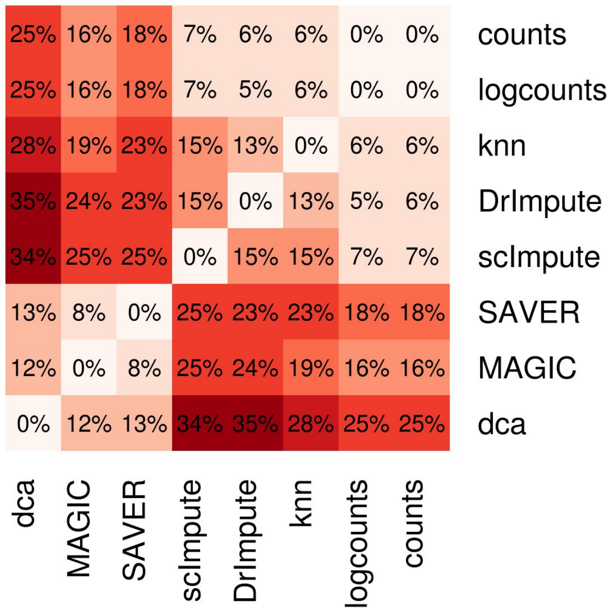

Figure S6: Contradictory marker directions between imputation methods. Proportion of significant markers (FDR 5%) that were assigned to different cell-types by different imputation methods in the same Tabula Muris dataset. Results were averaged across all Tabula Muris datasets.
