# Supplementary material for: False signals induced by single-cell imputation
Source: F1000Res. 2019 Mar 5;7:1740. Originally published 2018 Nov 2. [Version 2] doi: 10.12688/f1000research.16613.2 (PMC6415334; doi:10.12688/f1000research.16613.2)
Supplement: Peer review history

# Peer review: Reviewer response for version 2

**DOI:** 10.5256/f1000research.20056.r45285

**Published:** 2019-04-25

**Authors:** Simone Tiberi

**Affiliations:** 1 University of Zurich, Institute of Molecular Life Sciences, Zurich, Switzerland

The authors have addressed all the comments I made to Version 1.

 In my initial review I made numerous comments: I would like to thank the authors for their replies and for the time spent addressing them.

I have read this submission. I believe that I have an appropriate level of expertise to confirm that it is of an acceptable scientific standard.

# Peer review: Reviewer response for version 2

**DOI:** 10.5256/f1000research.20056.r45284

**Published:** 2019-03-15

**Authors:** Charlotte Soneson

**Affiliations:** 1 Friedrich Miescher Institute for Biomedical Research, Basel, Switzerland

In the revised version, the authors have nicely addressed all my comments from version 1. 

 There are a few places in the manuscript where the text should be updated to reflect the inclusion of an additional method, e.g. in the last paragraph of the Introduction.

 Also, the last sentence in the Background section of the Abstract seems to be missing a word (or should rather be split into two sentences), and the second sentence of the Results has the same passage repeated twice.

I have read this submission. I believe that I have an appropriate level of expertise to confirm that it is of an acceptable scientific standard.

# Peer review: Reviewer response for version 2

**DOI:** 10.5256/f1000research.20056.r45283

**Published:** 2019-03-12

**Authors:** Jean Fan

**Affiliations:** 1 Department of Biomedical Informatics, Harvard Medical School, Boston, MA, USA; 2 Department of Chemistry and Chemical Biology, Harvard University, Cambridge, MA, USA

The authors have done an excellent job addressing my concerns in this review. I have the following minor comments, primarily for clarification in the text:

 Comments:

- I find it very difficult to distinguish the colored lines used in the new Figure 2E and Figure S4 (in particular, the counts, log counts, and knn lines). Please change one of these lines to red or another more spectrally distinguishable color. For example, the colors in Figure 4 are easier to distinguish. 
- SAVER, MAGIC, and dca seem to introduce more contradictory markers compared to knn, DrImpute, and scImpute (Figure S6). Is this because of the underlying methodological biases discussed on page 9? Or should users be aware that using SAVER, MAGIC, and dca may introduce more contradictory differential expression results? Please clarify how users should interpret and act on these findings. 
- The authors note that filtering by effect size restores specificity of identified differentially expressed genes in the imputed data. Does this effect size filtering also fix the contradictory genes issue? Or are there still contradictory genes even after the effect size filtering? Please clarify. 

I have read this submission. I believe that I have an appropriate level of expertise to confirm that it is of an acceptable scientific standard.

# Peer review: Reviewer response for version 2

**DOI:** 10.5256/f1000research.20056.r45286

**Published:** 2019-03-11

**Authors:** Stephanie Hicks

**Affiliations:** 1 Johns Hopkins Bloomberg School of Public Health (JHSPH), Baltimore, MD, USA

The authors have addressed all of my comments from Version 1. Also, I want to thank the authors for their thoughtful responses to my comments.

I have read this submission. I believe that I have an appropriate level of expertise to confirm that it is of an acceptable scientific standard.

# Peer review: Reviewer response for version 1

**DOI:** 10.5256/f1000research.18156.r40239

**Published:** 2018-12-06

**Authors:** Stephanie Hicks

**Affiliations:** 1 Johns Hopkins Bloomberg School of Public Health (JHSPH), Baltimore, MD, USA

The authors Andrews and Hemberg provided an insightful analysis assessing whether or not false positives (or capturing false signals) are introduced by imputation methods into scRNA-seq data. Previous papers have only assessed true positives (or positive controls or ability to recover true signal). The authors considered both model-based (SAVER, DrImpute, scImpute) and smoothing-based (knn-smooth, MAGIC) imputation approaches where the former infers only the missing values and the latter smooths all the data (nonzero and zeros).

 I have a few suggestions and questions that I believe would help the manuscript: 

1. In the simulations (negative binomial and/or Splatter), my understanding is that the authors did not consider a simulation with batch effects (linear or non-linear, global effect or just a portion of the genes), and only considered dropouts in the Splatter simulation. An example with batch effects might be more realistic for scRNA-seq data from real biological experiments because batch effects have been shown to introduce false signals in data (Leek, 2010 ^1^). My concern is that the false positive signals reported here would actually be larger or more extreme in real scRNA-seq data. 
2. Could the authors explain the reason for using Bonferroni instead of Benjamini-Hochberg (BH) in correcting for multiple testing? I believe that BH is more commonly used in the context of high-throughput computational biology and genomics. Was it an intentional choice to impose a very conservative correction? Also, it would be interesting to use e.g. BH or even a more modern-controlling FDR methods (e.g. IHW from Wolfgang Huber's group). Hopefully this would only improve the ability to detect the true positives (e.g. positive controls), which leads me to my next question.
3. As sensitivity and specificity was considered in the Splatter simulations (Figure 2), could the authors show an ROC curve (e.g. averaged across the 60 scRNA-seq count matrices)? 
4. In the 'Permuted Tabula Muris datasets' section, the authors noted they used Euclidean distance as a form of similarity between two cell types. What about using correlation-based similarity measures instead of Euclidean which has been shown to be highly susceptible to the number of dropouts? 
5. For the approaches that were applied to the log2 transformed and normalized datasets, did the authors consider normalization methods specific for single-cell (e.g. scnorm or scran)? CPM has been shown to be not appropriate for scRNA-seq data (Vallejos *et al.,* 2017 ^2^), so I'm wondering if using a more appropriate normalization method improves the results any? 
6. I think one of the biggest concerns is the lack of reproducibility from certain imputation methods (as a side note, Figure 4C was confusing for me and I might suggest the authors consider illustrating this result a different way). This suggests more development is needed to make imputation methods more robust or an external dataset is needed (similar to using haplotype information for GWAS data). Could the authors comment on what they recommend? As this is a good example of a benchmarking paper comparing different imputation methods, I think it would be really useful for the authors to provide a set of recommendations for users. 

I have read this submission. I believe that I have an appropriate level of expertise to confirm that it is of an acceptable scientific standard, however I have significant reservations, as outlined above.

## References

[1] LeekJTScharpfRBBravoHCSimchaDLangmeadBJohnsonWEGemanDBaggerlyKIrizarryRA: Tackling the widespread and critical impact of batch effects in high-throughput data. *Nat Rev Genet*.11(10) : 10.1038/nrg2825 733-9 10.1038/nrg2825 20838408PMC3880143

[2] VallejosCARissoDScialdoneADudoitSMarioniJC: Normalizing single-cell RNA sequencing data: challenges and opportunities. *Nat Methods*.2017;14(6) : 10.1038/nmeth.4292 565-571 10.1038/nmeth.4292 28504683PMC5549838

# Response

**Published:** 2019-02-18

**Authors:** Tallulah Andrews

**Affiliations:** Wellcome Trust Sanger Institute

Thank you for the helpful suggestions, we have addressed all comments below and in the updated version of the manuscript: 

**1. **The Reviewer raises a very important point regarding batch effects. We agree that they are likely to make the situation worse for real datasets. However, they are still not well understood and differ greatly between studies in magnitude and genes affected making it difficult to simulate them well. We used Splatter to add small batch effects to all genes in our simulated datasets but this had relatively little effect on the imputation methods’ overall efficiency, however manual inspection of some of them showed that in some cases imputation methods can mistake batch effects for the real underlying structure. We have added this consideration to the Discussion.

**2.** This was a deliberate choice both (a) to be conservative and (b) to reduce the impact of imputation methods distorting the p-value distribution. We have clarified this in the text (Discussion: paragraph 4, Methods: Negative Binomial Simulations). This was specifically used for the Negative Binomial simulation as they did not mimic real single-cell datasets very well since they had many genes with very sharp differences between cell-types, and for testing the false-negatives in the permuted Tabula Muris datasets to avoid biases resulting from how the imputation methods affected genes that were actually differentially expressed in those datasets. For the splatter simulations and reproducibility of marker genes we used the more typical Benjamini-Hochberg/FDR correction since these better reflect real single-cell datasets and we were considering the ability to call true positives not specifically focusing on false positives. This has been clarified in the text (Methods: Splatter Simulations).

**3.** This was requested by another reviewer as well and we have added ROC curves to Figure 2.

**4. **We agree with the reviewer that Euclidean distance is susceptible to the number of dropouts. However, we only used the Euclidean distance only for picking which two cell-types to consider for the permutations thus has very little importance to our analysis, we could just as easily have picked cell-types at random, we only chose the two most similar to increase the number of genes that are not differentially expressed between the cell-types.

**5.** Only one method was designed to be run on already log2 transformed and normalized datasets (DrImpute), while several others (MAGIC, knn) internally apply CPM normalization. Thus, for consistency we used CPM for DrImpute. In addition, SCnorm is slow and scran frequently returns negative size factors unless one manually tunes its parameters for each dataset. Because of the high-throughput nature of our benchmarks we chose not to use these methods.

**6.** We have added recommendations for when and which imputation methods should be used to the Discussion (paragraph 5-6).

# Peer review: Reviewer response for version 1

**DOI:** 10.5256/f1000research.18156.r40875

**Published:** 2018-12-06

**Authors:** Charlotte Soneson

**Affiliations:** 1 Friedrich Miescher Institute for Biomedical Research, Basel, Switzerland

Andrews and Hemberg present an interesting evaluation of imputation and smoothing methods for scRNA-seq, focusing on false positive signals. Five recent imputation/smoothing methods are compared based on whether they: 

1. Introduce false correlations between genes in a Negative Binomial simulation without dropouts.
2. Accurately identify differentially expressed genes in simulated data with different degrees of dropout.
3. Induce false positives in differential expression analysis of permuted real scRNA-seq data.
4. Lead to reproducible sets of differentially expressed genes in data sets generated with different platforms.

The paper treats a relevant subject and is generally well written and easy to follow. Below are suggestions for clarifications and a few additions, which I feel would strengthen the paper and provide additional guidance for the reader in determining which, if any, method to use.

 Major comments:

1. As the authors note, the evaluated methods are based on different distributional assumptions. Since the goal of the imputation is to retrieve the "true underlying signal", performance is likely to be strongly affected by the distribution of the data used for evaluation. In the evaluation of falsely induced correlations (a), it would thus be informative to consider different plausible distributions (not only the Negative Binomial), and compare the performance of the methods. In order to avoid making distributional assumptions, perhaps an appropriate bulk RNA-seq data set could also be useful at this stage. 
2. It would be useful to explicitly spell out the underlying models used by each of the methods, as well as the type of input that they were provided with (raw counts or log-transformed normalized values) and the scale of the output (count or log-count scale) in Table 1. I was also wondering whether correlations in (a) were always calculated on the count scale, or whether they were calculated on the log-scale for some methods. It might be useful to also show the correlations with unimputed log-transformed data in Figure 1A, since not all cells have exactly the same library size/size factor.
3. Depending on the type of protocol used for the library preparation, scRNA-seq data could have different distributional properties. Since the authors include both SmartSeq2 and 10x data, it would be interesting to see a discussion of the relative merits of the different methods related to the platform used to generate the data. In particular, I was wondering what type of data that the Splatter simulations most resemble, and whether simulations similar to different types of scRNA-seq data could be generated. It would be helpful to see a comparison of the main characteristics of the simulated data and those of real scRNA-seq data, to know to what extent the conclusions drawn from the simulations can be expected to be generalizable to real data sets.
4. No attempt is made at explaining the large differences between the Tabula Muris tissues in terms of the number of false positives in the permuted data. Are there any apparent differences between the data sets that might (at least partly) explain this? I think it would also be useful to include the results from unimputed data in Figure 4A-B.
5. Given that there are already several imputation/smoothing methods available that were not explicitly evaluated in this study, and that it is likely that this number will increase quickly, it would be very useful if the evaluation would be easily extendable. As a minimum, it would be useful to make the code available, preferably structured in a modular way so that new methods can be easily substituted. Depending on the time and effort required to generate and process the data sets, these could also be made available.

Minor comments:

1. It is not immediately clear what the numbers in the "Dropouts (midpoint)" column in Table 2 represent. 
2. I think it would be worth briefly mentioning Figure S1 in the text, rather than just referring to it in the caption of Figure 1, without discussing its content further. 
3. For the reproducibility evaluation, only the number of significant genes shared between SmartSeq2 and 10x are reported. How many genes were found to be significant in one data set only?
4. The panels in Figure 5 would be easier to compare if the y-axes were the same.
5. There are a few typos and inconsistencies (e.g., knn-smooth/knn smooth, raw-counts/raw counts, Smart-seq2/Smartseq2, cell-types/cell types) throughout the text.
6. It is not always clear how the statistical tests were applied. For the count-scale data, were the values somehow normalized between cells before the tests were applied? Also, for the log-normalization of the data, what pseudo-count was used, and how were the size factors calculated?

I have read this submission. I believe that I have an appropriate level of expertise to confirm that it is of an acceptable scientific standard, however I have significant reservations, as outlined above.

# Response

**Published:** 2019-02-18

**Authors:** Tallulah Andrews

**Affiliations:** Wellcome Trust Sanger Institute

Thank you for the helpful suggestions, we have made all suggested Minor corrections and have addressed the Major corrections here and in the revised version of the text:

**1. **It has been established that read counts from scRNA-seq and bulk RNA-seq (or indeed other -seq protocols) are well described by some variant of the negative binomial distribution e.g. (Grün *et al.* 2014; Robinson and Smyth 2007), which is why that is the model used here for the simulations. We have added Figure S1 to the supplementary material showing the Splatter simulations are a good match for real scRNA-seq data. However, it should be noted that we find that 10X data was best simulated as a pure negative binomial, whereas Smart-seq2 was best simulated with a zero-inflated negative binomial as has been remarked upon previously (see: http://www.nxn.se/valent/2017/11/16/droplet-scrna-seq-is-not-zero-inflated). In addition, when comparing the fits of the zero-inflated negative binomial distribution and zero-inflated normal distribution to the Tabula Muris raw counts and log-normalized counts respectively we found the negative binomial fits the vast majority of genes better than the normal distribution (Table S1). Thus, we believe the negative binomial to be the most sensible distribution for simulating scRNA-seq data.

While we agree bulk RNA-seq intuitively seems like a good ‘ground truth’ for scRNA-seq it is difficult to use it to evaluate imputation since in general simply summing scRNA-seq data is the closest approximation to bulk RNA-seq by the nature of the experiments. The use of bulk RNA-seq as a ground truth assumes that the assayed cell-populations are in truth completely homogeneous. If the “pure” cell populations are a result of sorting this is almost certainly not correct because there is always a fraction of contaminating cells which will result in a bias towards greater smoothing. Although cell populations obtained by growing cells in culture are more likely to be homogenous, they are a poor model for scRNA-seq data obtained from complex tissue samples. There are also reasons to believe that bulk RNA-seq is not a gold standard for identifying truly differentially expressed genes. Bulk RNA-seq is generally limited by its low power due to a small number of samples and the homogenizing effect of bulk samples. Thus, genes that are simply not-detected as differentially expressed using bulk RNA-seq may in truth be differentially expressed just in a small subset of cells or with a low fold-change. Moreover, even though there are many common steps in the experimental protocols for generating bulk and scRNA-seq, it is likely that there will be effects that are specific to each method. For example: with respect to GC content biases or gene-length biases, bulk RNA-seq may not be more correct than scRNA-seq. There is no reason to believe reproducibility across bulk and scRNA-seq is a more reliable method of benchmarking than reproducibility across different scRNA-seq datasets which we have performed using the Tabula Muris data.

**2.** We thank the Reviewer for this suggestion. We have added information about the input, output and underlying model  to Table 1 and we have also clarified in the Methods how the correlations were calculated.We have also added the unimputed log-transformed data to Figure 1A.

**3.** We have added Figure S1 comparing the general properties of the real Smart-seq2 and 10X datasets with the Splatter simulations. Generally they are a good match, though the 10X data more closely resemble data simulated with few/no added dropouts, whereas the Smart-seq2 data more closely resembles data with relatively high numbers of added dropouts. 

In another recent publication from the group (Westoby *et al.* 2018, Genome Biology), we carried out extensive simulations for comparing isoform quantification methods. We concluded that the splatter simulations did a very good job at resembling the Smart-seq2 data, but the comparisons to Drop-seq data were more tenuous (the discussions on the Drop-seq data were removed from the final version but can be found in the Biorxiv version). 10x data closely resembles Drop-seq data, so those conclusions are likely to hold.

**4.** This was also requested by another reviewer and we have included the unimputed data in Fig 4A and B. We considered the diversity of cell-types, average sequencing depth, number of detected genes, and number of cells, and the goodness of fit of genes to a zero-inflated negative binomial distribution (in table S1) as possible explanation for the variability between datasets but none of them were particularly associated with number of false positives by different methods. However manual inspection of the effect of imputation on the Tabula Muris data (Figure S4) suggests the variable performance across datasets is related to biases in correcting for library size, which would be a combination of differences in cell-size and degree of difference (DE) between cell-types.

**5.** We have made the scripts in a modular structure for the comparison available on github. Thus, it should be straightforward to add methods and re-run the study.

# Peer review: Reviewer response for version 1

**DOI:** 10.5256/f1000research.18156.r40894

**Published:** 2018-12-03

**Authors:** Jean Fan

**Affiliations:** 1 Department of Biomedical Informatics, Harvard Medical School, Boston, MA, USA; 2 Department of Chemistry and Chemical Biology, Harvard University, Cambridge, MA, USA

Overview:

 Analysis of single-cell RNA-seq data is often complicated by large amounts of zeros, of some which represent true lack of expression, while others are reflective of poor capture efficiency or other technical limitations. Several methods have been developed to impute the zeros and recover the true gene expression values. Here, Andrews and Hemberg compare the performance of 5 of these single-cell imputation methods using both simulated data and artificially permuted single-cell RNA-seq data. They evaluate the extent to which these methods introduce false differential expression. A number of clarifications are needed to improve the understandability of the manuscript. Performance benchmarks using additional datasets are also needed to ensure that observed performance differences between methods are not biased by how well the datasets conform to underlying distributions assumed by each method. 

 Major comments:

1. The authors conclude that SAVER is the least likely to generate false positives and should be favored over the other 4 imputation methods. However, the scImpute manuscript compared its performance with SAVER to draw conflicting conclusions. I am concerned that the conclusion of which method is better is being biased by the way the benchmark data has been simulated in both cases. Here, the authors simulate data using a negative binomial distribution and find that SAVER had the lowest false positive rate. However, as the authors note, this may be expected, since SAVER models expression data using a negative binomial model. In this manner, the simulation results appear rather circular: the method that uses the same model as the simulated data performs best. In contrast, in the scImpute paper, the authors simulate data using a normal distribution with drop-outs introduced using a Bernoulli distribution and find that SAVER imputation does not alter the data by much or improve downstream clustering whereas scImpute recapitulates the complete data. Please discuss this discrepancy. 
2. There are genes that are not detected in most single-cells due to poor capture efficiency but we know must be expressed, albeit at low levels, based on bulk RNA-seq, FISH, RT-qPCR, or other approaches for measuring gene expression. As a result, most previous methods have assessed performance by comparing imputed values from single-cell RNA-seq against these bulk RNA-seq, FISH, or RT-qPCR datasets, typically focusing, as the authors note, on the imputation method's ability to recover true signals. How often does imputation introduce a significantly differentially expressed gene in single-cell data that we know should not be differentially expressed based on bulk RNA-seq, FISH, RT-qPCR, or etc? Bulk RNA-seq and single-cell RNA-seq datasets exist for both ESC and DEC cells, which were used for benchmarking in the scImpute paper. Both sorted and unsorted PBMCs are also widely available in both bulk and single-cell RNA-seq form. A number of cell lines have also been sequenced by both bulk and single-cell RNA-seq. In general, the manuscript would greatly benefit from the inclusion of additional benchmarks based on at least one of these datasets. Including additional datasets will also help mitigate the concern that SAVER's superior performance over the other methods is simply the result of both the simulated and the Tabula Muris dataset conforming to the negative binomial model. 
3. The authors find that many randomly permuted genes were differentially expressed after imputation and furthermore, the direction of the differential expression after imputation was different for different imputation methods. How frequently do these different imputation methods lead to these different directions of differential expression and therefore conflicting biological interpretations? Is Zfp606 the only gene that exhibits this issue suggesting this is a rare event? Or do conflicts arise frequently?
4. The authors identify marker genes prior to imputation and note that 95% of marker genes are significant markers in both SmartSeq2 and 10X datasets for the same tissues. They use this comparison between SmartSeq2 and 10X datasets to quantify reproducibility. After imputation, only 80% or so of marker genes were significant in both datasets i.e. decreased reproducibility. Is this decreased reproducibility just due to significance thresholds being reached in one dataset but not the other? Are the -log10(p-values) from the Mann-Whitney-U tests correlated before and after imputation? How do the -log10(p-values) from the Mann-Whitney-U tests correlate between SmartSeq2 and 10X? Before and after imputation?

Minor comments:

1. The terms "false positive", "false signal", and "false positive signal" are used throughout the early components of the manuscript, including the abstract, before it is defined in the "Permuted Tabula Muris datasets" section. I initially interpreted "false positive signal" loosely to mean genes that are not supposed to be expressed but become non-zero after imputation. However, the definition that the authors are using appears more stringent in that not only does a gene become non-zero after imputation but it becomes significantly differentially expressed. I appreciate this more stringent definition since it more directly impacts biological interpretation. Please define "false positive signal" earlier or use a more specific term like "false differential expression" to minimize confusion due to terminology. 
2. The terms "irreproducible results", "reproducibility", etc. are used throughout the early components of the manuscript, including the abstract before it is defined in the "Reproducibility of markers" section. I initially interpreted "reproducibility" to mean whether I would get the same results from running the same imputation algorithm multiple times.  Please define these terms earlier or use a more specific term to minimize confusion due to terminology. 
3. The authors note that many imputed markers were assigned to "contradictory cell-types" (page 8). Please clarify what this means. What fraction of identified markers does this affect? Does this tend to affect one cell-type i.e. are the markers consistently mixed up between two cell-types?  
4. Please clarify which methods were run on raw counts and which were run on log2 CPM in Table 1. Was a pseudocount used in the log transformation? 
5. The authors state that "scRNASeq imputation only draws on structure within the dataset itself" but this statement should be limited to the scope of the 5 methods currently tested. scRNAseq imputation methods in the future may draw on external datasets. 
6. Figure 1A is very telling. Could a similar figure be included for the Tabula Muris datasets to visualize the effects of imputation?
7. Readers would greatly benefit from a discussion on when imputation should be used, if at all, given this observed propensity to introduce false differential expression.

I have read this submission. I believe that I have an appropriate level of expertise to confirm that it is of an acceptable scientific standard, however I have significant reservations, as outlined above.

# Response

**Published:** 2019-02-18

**Authors:** Tallulah Andrews

**Affiliations:** Wellcome Trust Sanger Institute

Thank you for the helpful suggestions, we have made all suggested Minor corrections and have addressed the Major corrections here and in the revised version of the text:

**1.** Our results broadly agree with the results presented in the scImpute paper, in that SAVER makes modest adjustments to the data, and MAGIC introduces many false signals, whereas scImpute falls in between. However, the scImpute paper focuses on the ability of the method to amplify true signals, such as the tightness of clusters, and the strength/detection of true differential expression, within the data. Whereas, our analysis focused on the tendency to introduce false-positives. Thus it provides complementary rather than contradictory information. 

Since scImpute uses a zero-inflated normal distribution to approximate log-transformed normalized counts it is expected that it would outperform other methods when that model is used for the simulations as in the scImpute paper. However, RNA-seq data is fundamentally a discrete non-negative process, thus violating the assumptions of the normal distribution. It has been established that read counts from scRNA-seq and bulk RNA-seq (or indeed other -seq protocols) are well described by some variant of the negative binomial distribution e.g. (Grün *et al.* 2014; Robinson and Smyth 2007), which is why that is the model used here for the simulations. We have added Figure S1 to the supplementary material showing the Splatter simulations (zero-inflated negative binomial) are a good match for real scRNA-seq data. However, it should be noted that we find that 10X data was best simulated as a pure negative binomial, whereas Smartseq2 was best simulated with a zero-inflated negative binomial as has been remarked upon previously (see: http://www.nxn.se/valent/2017/11/16/droplet-scrna-seq-is-not-zero-inflated). In addition, when comparing the fits of the zero-inflated negative binomial distribution and zero-inflated normal distribution to the Tabula Muris raw counts and log-normalized counts respectively  we found the negative binomial fits most genes better than the normal distribution (Table S1). Thus, we believe the negative binomial based simulations used here are more relevant to real single-cell RNA-seq data than the simulations used in the scImpute paper.

**2.** While we agree bulk RNA-seq intuitively seems like a good ‘ground truth’ for scRNA-seq it is difficult to use it to evaluate imputation since in general simply summing scRNA-seq data is the closest approximation to bulk RNA-seq by the nature of the experiments. The use of bulk RNA-seq as a ground truth assumes that the assayed cell-populations are in truth completely homogeneous. If the “pure” cell populations are a result of sorting this is almost certainly not correct because there is always a fraction of contaminating cells which will result in a bias towards greater smoothing. Although cell populations obtained by growing cells in culture are more likely to be homogenous, they are a poor model for scRNA-seq data obtained from complex tissue samples. There are also reasons to believe that bulk RNA-seq is not a gold standard for identifying truly differentially expressed genes. Bulk RNA-seq is generally limited by its low power due to a small number of samples and the homogenizing effect of bulk samples. Thus, genes that are simply not-detected as differentially expressed using bulk RNA-seq may in truth be differentially expressed just in a small subset of cells or with a low fold-change. Moreover, even though there are many common steps in the experimental protocols for generating bulk and scRNA-seq, it is likely that there will be effects that are specific to each method.  For example: with respect to GC content biases or gene-length biases, bulk RNA-seq may not be more correct than scRNA-seq. There is no reason to believe reproducibility across bulk and scRNA-seq is a more reliable method of benchmarking than reproducibility across different scRNA-seq datasets which we have performed using the Tabula Muris data. We attempted to use two datasets (Kolodziejczyk *et al.* 2015; Tung *et al. *2017) for which matching bulk data was available but the results were inconsistent which is not surprising considering the variability we saw with the Tabula Muris datasets.

**3.** We have added Figure S6 which shows that the proportion of markers with conflicting directions across all the Tabula Muris datasets varies from 5% to 35%. We considered the full imputed Tabula Muris dataset since most genes should have some real differential expression, and thus be more likely to be consistent across imputation methods than the permuted genes, which contain no true signal.

**4.** We apologize that this analysis was not explained clearly. The 95% and 80% are not related to differences in power or significance thresholds, they refer to the percent of markers that were most highly expressed in the same cell-type given that the gene was a significant marker in both datasets. We have clarified this in the text (Results: page 13-14). We appreciate the suggestion for comparing p-values directly and have added a supplementary figure S5 that displays these correlations, further reinforcing our original conclusions that imputation results in poorer reproducibility.

# Peer review: Reviewer response for version 1

**DOI:** 10.5256/f1000research.18156.r40895

**Published:** 2018-11-29

**Authors:** Simone Tiberi

**Affiliations:** 1 University of Zurich, Institute of Molecular Life Sciences, Zurich, Switzerland

The article investigates how imputation methods of 0 counts in single-cell RNA-seq (scRNA-seq) can introduce false signals, and hence false positives in downstream analyses. The authors explain how scRNA-seq data can present an excess of 0 counts, i.e. dropouts, due to technical artefacts, and introduce a few recent methods that can be used to impute these values. Andrews and Hemberg focus on a sub-set of 5 imputation methods and investigate, in three scenarios, if these methods introduce false signals between genes:

1. First, data are simulated from a simple negative binomial (NB) model: most imputation methods introduce false signals in the data by increasing the correlation between independent genes.
2. Secondly, the authors study the effect of imputation methods on downstream differential gene expression (DGE) analyses on 60 scRNA-seq datasets simulated via Splatter (with varying degrees of dropouts and DGE). They find that, compared to the original un-imputed data, albeit some imputation methods result in higher Sensitivity (i.e. true positive rate), all of them have lower the Specificity (i.e. true negative rate).
3. Thirdly, they consider several real scRNA-seq datasets, where counts are permuted to obtain approximately uncorrelated genes, and investigate how imputation methods affect the ability to identify marker genes. The authors find that, compared to the un-imputed data, imputation tools distort expression patterns and increase the number of identified marker genes, although some of these are likely to be false detections.

The article treats a relevant problem and provides a comprehensive benchmarking of imputation methods. Overall, the manuscript is clear and its scientific quality is adequate. Below, I suggest several corrections (and identify a few typos) that hopefully will contribute to improving the quality and clarity of the work.

**Major Comments:**

1. In some cases it is unclear to me why you take certain decisions: I feel you should motivate more your choices (see Minor comments for specific examples).
2. Please provide source code to reproduce all the analysis you present (including obtaining the simulated and permuted data).
3. There is some redundancy in the description of the data: you first describe in detail how you obtained the simulated data and the permuted real data in the Methods section, and then you repeat it again (although with fewer sentences) in the Results section. I would avoid or shorten the second description in the Results section.
4. Although the paper aims at investigating on false signals introduced by imputation methods, I feel too much emphasis has been given to false positive results as opposed to jointly considering false and true positive results. Indeed, the paper shows that imputation methods result in increased FPs/Specificity, particularly when the original data are not affected by dropouts, but it only marginally focuses on the increase in TPs/Sensitivity.
  More informally, I think you should try to show both sides of the coin and avoid (over-)interpreting FP results alone. In this regard, to get a joint picture of Sensitivity and Specificity, I think you should provide (at least for the Splatter simulation) ROC and FDR curves (eventually, also as Supplementary figures). Since you perform 60 simulations from Splatter, you might consider global ROC and FDR plots based on the results from all simulations.
5. I think that the limitations of the study should be explained more clearly:
  5.1) In the permuted real data analysis, all imputation methods find many more marker genes than the un-imputed data, but the authors mostly focus on the fact that the percentage of “reliable” identifications decreases. I think that: 1) importance should be given also to the fact that many more “reliable” marker genes are identified (also referring to the comment above about FPs and TPs) and: 2) it is essential to explicitly acknowledge that the true state of marker genes is unknown. Importantly, in Figure 4 A) and B) please add the FPR obtained on the un-imputed data to provide a baseline comparison.
  5.2) In the NB simulation you don’t simulate any dropouts, which represents the worst case scenario for imputation methods. In this context, I would expect all imputation methods to worsen downstream results, because there are no dropouts to impute at all. I think you should mention this more explicitly.
6. In Splatter simulations you “considered the effect of four different amounts of added dropouts”. How mild or extreme were these dropout levels compared to real scRNA-seq data? I would expect imputation methods to improve the quality of the data as the number of dropouts increases. Did you try to consider more “extreme” dropout rates?
7. In Figures 2C and 2D you provide Sensitivity boxplots stratified by dropout rates and Specificity boxplots stratified by DE. Sensitivity and Specificity should always be examined jointly: for both stratification cases, please provide both Sensitivity and Specificity plots (eventually, also as Supplementary figures).
8. I suggest another round of polish to improve writing and clarity in some parts of the paper. In particular: adding few commas would facilitate the reading in long sentences; past and present tenses are sometimes mixed; some sentences seem a bit out of place and could be better integrated in the flow; I found the last two paragraphs of the Results section a bit hard to follow.
9. You refer a few times to the fact that you “find a fundamental trade-off between sensitivity and specificity which imputation cannot overcome”: reading the paper it seems that imputation methods might be responsible for this. But this trade-off is due to the nature of Sensitivity and Specificity; indeed, Sensitivity and Specificity are positively correlated by construction: as one moves the significance threshold, both will increase or decrease. Clearly an ideal method will have Sensitivity 0 and Specificity 1. I think you should remove or edit the sentences referring to this trade-off (particularly in the Discussion) to clarify that imputation methods are not the cause of this trade-off.
10. In the Discussion you say that “While imputation in other fields often uses external references or relationships for the imputation, scRNASeq imputation only draws on structure within the dataset itself.”. Actually, “canonical” imputation methods do not require an external reference and only use the available data. While having an additional reference can increase the information at disposal and hence, potentially, improve the accuracy of imputation tools, I don’t think this is the main reason why they result in increased false signals. Besides, there are other issues with using an external reference; e.g. if the reference is not “similar” to the data-set under study, particularly concerning their dropouts. I think you could clarify that using an external reference is one of the possible ways to improve imputation methods, but keeping in mind that imputation (in general) can also work without a reference.

**Minor Comments:**

 1) General:

- Throughout the text, you use both “Smart-seq2” and “Smartseq2“; I suggest you use only one, for consistency.

2) Abstract:

- “since these methods generally rely on structure inherent to the dataset under consideration they may not provide any additional information.” You clarify this point later in the text but, when I read the abstract, it was not clear to me what you were referring to. Maybe you could try to be more explicit here or remove the sentence.

3) Introduction:

- You cite 4 imputation methods as “under development“ but you only test one. I think you’d motivate this choice.
- Typo: “though imputation” -> “through imputation”.
- GWAS not defined before.
- Typo: “imputation, which only attempt to infer” -> “imputation, which only attempts to infer”.

4) Methods:

- Fig S1: “aka” -> “i.e.” (I would use something more elegant than aka).
- Typo: “as calculated scater” -> “as calculated by scater” ?
- “ranging from 10^3-10^4” -> “ranging 10^3-10^4” or “ranging from 10^3 to 10^4”.
- Typo: “different probability distribution” -> “different probability distributions”.
- “When filtering DE genes by effect size, in addition to significance”. This sentence is quite vague, please be more specific.
- “Six 10X Chromium and 12 Smartseq2 datasets”. You use words (Six) and digits (12) in the same sentence to refer to numbers: I’d choose one for consistency.
- You use two distinct types of DGE tests for the simulated data (Splatter) and the permuted real data. Please motivate your choice.
- Typo (?): “for which there exists matching Smart-seq2 and 10X Chromium” -> “for which there exists matching for Smart-seq2 and 10X Chromium” ?

5) Results:

- “MAGIC provides  … whereas knn smooth provided …”. Present and past tenses are mixed here: I suggest you replace “provided” with “provides” to keep consistency with the rest of the manuscript.
- In the NB simulation, provide more details on the implementation of the correlation test: how did you test correlations? What significance level was used to define a significant correlation in Fig 1B and S1? 0.05?
- In Figure 1B and S1, I guess that “Raw” refers to the original (un-imputed) data; did I understand correctly? It was not obvious to me at a first glance, please make it explicit (in the text or in the Figure caption).
- Fig 2: typo (?): “Different imputation methods choose a different trade-off ...“;  I didn’t understand the use of “choose” in the sentence: is this a typo? If not, can you re-write the sentence in a clearer way?
- Fig 2: “genes DE” -> “DE genes”.
- In the permutation real data analysis, please clarify the concept of filtering genes: do you refer to independent filtering of genes (based on their estimated FC)?
- Typo: “the bulk of false-positives ... result” -> “the bulk of false-positives … results”.
- “It’s possible” -> “It is possible”.
- “Xth percentile” -> “X-th percentile”.
- “Xth percentile highest log2 fold-change“ -> “highest log2 fold-change X-th percentile”.
- Fig 4 (A) caption: “SmartSeq2 datasets,” -> “SmartSeq2 datasets.” (a comma separates two Figure descriptions instead of a full stop).
- Fig 4 (C) caption: “the proportion that were markers” -> “the proportions that were markers”.
- I would change “many of the imputed markers are incorrect” to “some of the imputed markers are incorrect”. “some” seems more appropriate than “many”, considering that 80-90% of them are estimated to be true marker genes.
- The second last paragraph of Results sounded a bit contorted to me: I would rephrase it in a clearer way.
- “The imputation methods produced different distortions of the gene expression values ( Figure 6).” Can you better integrate this sentence in the flow? It seems a bit out of place.
- “PCA and differential expression” -> “PCA and most differential expression tools/methods”. Tools/methods is missing. I would also add “most” because not all DE methods require NB or Gaussian distributions (e.g. non parametric methods).
- To facilitate a visual comparison, in Figure 5 I would adjust the left y-axis (Genes #) to have the same limits in all examples.
- Fig 6 caption: “significant after Bonferroni correction”; please add the significance level (I assume 0.05).

6) Discussion:

- In the second paragraph you first use “these methods generate” and then “MAGIC generated” mixing present and past tenses; I’d use “generate” in both cases.
- The subject is missing in this sentence: “MAGIC and knn-smooth which are data-smoothing methods, as such they adjust all expression values not just zeros.” -> I would write something like: “MAGIC and knn-smooth are data-smoothing methods, as such they adjust all expression values not just zeros.” Or alternatively, “MAGIC and knn-smooth, which are data-smoothing methods, adjust all expression values not just zeros.”
- “it’s performance” -> “its performance”.

I have read this submission. I believe that I have an appropriate level of expertise to confirm that it is of an acceptable scientific standard, however I have significant reservations, as outlined above.

# Response

**Published:** 2019-02-18

**Authors:** Tallulah Andrews

**Affiliations:** Wellcome Trust Sanger Institute

Thank you for the helpful suggestions, we have made all suggested Minor corrections and have addressed the Major corrections here and in the revised version of the text:

**1.** We have revised the text and tried to provide motivations for the key decisions.

**2.** The github repo accompanying the study now contains scripts that can be run to reproduce the results reported here.

**3.** We have followed the Reviewer’s suggestion and shortened the descriptions in the Results section.

**4.** We have added the true positive rate to Figure 1B, have added Figure S4 showing the increase in reproducible markers in Tabula Muris datasets, and modified the text to put greater emphasis of the increase in sensitivity provided by imputation (last paragraph of page 6, p9 paragraph 2, p14 paragraph 1) to clarify that sensitivity is increased by using imputation at the cost of specificity, however as the ROC plots show (Figure 2 E), to address the reviewer’s concern below, this increase in sensitivity could be achieved by simply lowing the significance threshold applied to the statistical test and result in fewer false positives than using an imputation method.

The reviewer raises a good point and we have calculated and included the ROC for the simulated data in Figure 2 E.

**5.1.**

1. We have updated the text to highlight the advantage of having a larger number of markers from imputed data (Figure S4, p14 paragraph 1).
2. We have added the FPRs for the un-imputed data (counts) to both Fig 4A and B, as expected there were almost none since we used the conservative Bonferroni multiple testing correction.

**5.2.** We have highlighted the lack of dropouts in the NB simulations in the text, and explicitly mentioned the desired behaviour for both model-based imputation and data-smoothing in this context (Methods: Negative Binomial Simulations).

**6.** We have adjusted the dropout parameters tested to be more similar to those observed in real single-cell RNA-seq data (Figure S1 A) and added the average proportion of zeros in the entire expression matrix for each value to Table 2 to help the readers understand what the different scenarios correspond to. At the highest level of added dropouts 94% of the matrix was composed on zeros and all the methods other than MAGIC and knn-smooth had sensitivity < 0.2, and the resilience of data-smoothing to high dropout rates has been noted in the text (Results: p9, paragraph 1).

**7.** We have followed the Reviewer’s suggestion and now include both Sensitivity and Specificity plots stratified by dropout rate and proportion of DE genes in Figure 2.

**8.** We have tried to improve the clarity of the text with a specific focus on paragraphs highlighted by the Reviewer.

**9.** The Reviewer raises an important point regarding the fundamental relationship between sensitivity and specificity. One of the central aims of our paper was to highlight this particular trade-off and that the effect of most imputation methods is simply to shift the balance between these quantities. Our goal was to say that this is indeed a relationship that is caused by how these quantities are constructed and that imputation methods simply favour one side of the trade off or the other not create it. We have edited the text to better clarify this (Discussion: paragraph 1).

**10.** The Reviewer raises a good point, we have edited the text appropriately (Discussion: paragraph 3).
